# pH-responsive injectable hydrogel dressing integrating antibacteria, antioxidation, anti-inflammation, and angiogenesis for infected wound healing

**DOI:** 10.1016/j.ijpx.2026.100591

**Published:** 2026-06-27

**Authors:** Ziwei Wang, Hongxia Zhao, Longxuan Zhan, Yifei Zhang, Yongzhe Liu, Xiaoni Ma, Baojin Ma

**Affiliations:** Department of Implantology & Tissue Engineering and Regeneration, School and Hospital of Stomatology, Shandong University & Shandong Key Laboratory of Oral Tissue Regeneration & Shandong Engineering Research Center of Dental Materials and Oral Tissue Regeneration & Shandong Provincial Clinical Research Center for Oral Diseases, Jinan 250012, China

**Keywords:** Immune regulation, Injectable hydrogels, Accelerate infected wound repair, pH-responsive

## Abstract

Infected wounds have presented a significant clinical challenge due to persistent inflammatory responses, elevated oxidative stress, and damaged blood vessels, which markedly impede the healing process. Here, we develop a multifunctional hydrogel dressing (named SMCC) based on sodium alginate, co-loaded with minocycline and curcumin, which exhibits pH-responsive characteristics. This hydrogel exhibits excellent injectability and adaptability, enabling it to conform to irregular wound surfaces and facilitate accelerated drug release in acid microenvironments, thereby synergistically exerting antibacterial, anti-inflammatory, and antioxidant effects. *In vitro* experiments demonstrate that SMCC hydrogel exhibits outstanding biocompatibility, effectively promotes cell migration, and enhances angiogenesis by upregulating VEGF and CD31 expression. Furthermore, the hydrogel significantly mitigates H_2_O_2_-induced oxidative stress by scavenging excess ROS. Importantly, SMCC hydrogel downregulates pro-inflammatory factors while upregulating anti-inflammatory markers, thereby inhibiting M1 polarization and promoting M2 polarization of macrophages. In rat models of full-thickness infected skin defect, SMCC hydrogel effectively remodels the inflammatory microenvironment, enhances vascularization and collagen deposition, and consequently accelerates wound healing and tissue regeneration. This study provides a promising functionalized hydrogel-based strategy for the comprehensive management of infected wounds.

## Highlights

• Infected wound challenges due to existing bacteria and elevated oxidative stress.
• SMCC hydrogel releases minocycline and curcumin in a pH-responsive manner.
• SMCC exerts synergistic antibacterial and antioxidant effects by scavenging ROS.
• SMCC accelerates healing by enhancing angiogenesis and inflammatory remodeling.

## Graphical abstract

**Figure:**
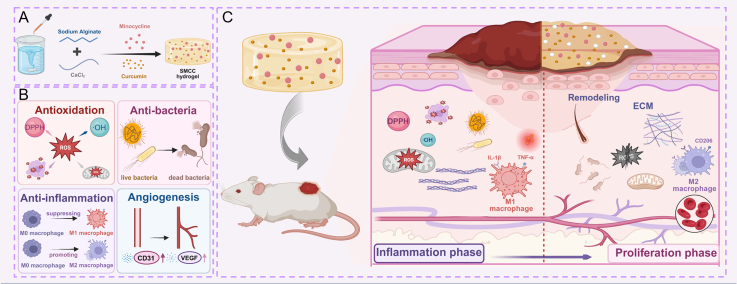
Schematic illustration of the synthesis of the SMCC hydrogel with multifunctional properties for infected wound healing and skin reconstruction.

## 1 Introduction

As the largest organ of the human body, the skin functions as a critical physical and immunological barrier against microbial invasion and environmental damage (Wu et al., 2023). Infected injuries are frequently complicated by persistent infection, sustained inflammation, and impaired healing, posing a major clinical and socioeconomic burden worldwide (Zhao et al., 2024). Wound healing is a tightly regulated, multi-phase process comprising hemostasis, inflammation, proliferation, and remodeling, each driven by specific cellular and molecular mechanisms. Disruption of these orderly progressions can lead to delayed wound healing (Wells et al., 2016; Fakher and Westenberg, 2025). A hallmark of infected wounds is prolonged inflammation, characterized by persistent and excessive production of reactive oxygen species (ROS) (Kant et al., 2014). Elevated levels of pro-inflammatory cytokines, including IL-1β and TNF-α, further extend the inflammatory phase and delay tissue repair (Beidler et al., 2009). These cytokines also upregulate matrix metalloproteinases (MMPs), leading to excessive degradation of the extracellular matrix (ECM) and impaired cell migration (Tarnuzzer and Schultz, 2002; Soneja et al., 2005; Shi et al., 2023). Concurrent oxidative stress contributes to DNA damage, lipid peroxidation, and enzyme inactivation, which collectively aggravate inflammation and hinder remodeling (Mohanty et al., 2012). Moreover, the presence of bacterial biofilms further exacerbates the condition by shielding microorganisms from immune clearance, thereby sustaining a high microbial burden that perpetuates inflammation and disrupts healing (Otto et al., 2013; Roche et al., 2012).

Conventional dressings, such as gauze, are generally inadequate in managing the complex microenvironment of infected wounds, which involves infection, unresolved inflammation, and impaired vascularization (Casadidio and Censi, 2025). Their limitations frequently result in delayed healing, increased healthcare costs, and reduced quality of life. In contrast, hydrogel dressings derived from natural polymers, such as alginate, hyaluronic acid, and gelatin, have emerged as promising wound dressings due to their high biocompatibility, biodegradability, low immunogenicity, and exceptional moisture-retention capacity. The hydrophilic three-dimensional network of hydrogels conforms to the wound bed, provides a barrier against bacteria, and maintains a moist environment that supports cellular regeneration and wound closure (Yuan et al., 2023; Sang et al., 2024; Wang et al., 2023).

The pH value at the wound site is a key biological indicator of tissue healing status, with its fluctuations directly modulating essential repair processes, including inflammatory response, collagen synthesis, and angiogenesis, which vary dynamically throughout the healing stages (Cui et al., 2022). In the infected sites, localized vasoconstriction and impaired blood supply result in nutrient and oxygen deficiency, enhanced anaerobic glycolysis, and lactic acid accumulation, collectively establishing an acidic milieu (pH = 5.2–6.5) (Zha et al., 2023; Han et al., 2024). Therefore, hydrogel structure changes by a pH-responsive mechanism can serve as a crucial signal for triggering drug release in such an environment (Uddin et al., 2024).

Alginate, a linear anionic polysaccharide derived from brown algae or bacteria, consists of varying proportions of β-1,4-linked D-mannuronic acid (M) and L-guluronic acid (G) residues (Varaprasad et al., 2020). Its ability to form crosslinked structures, most notably the “egg-box” complex with divalent cations such as Ca^2+^ *via* G-unit interactions, makes it particularly suitable for biomedical applications. Owing to its high absorbency, hydrating capacity, degradability, and minimal irritancy, sodium alginate (SA) has been widely employed in wound management, especially for exudative wounds. It supports moist wound healing, allows pain-free dressing changes, and serves as a base material for films, hydrogels, and nanofibers. However, its relatively poor mechanical properties and lack of inherent antibacterial activity limit its broader therapeutic utility (Zhang et al., 2021; Zamani et al., 2024).

To address these shortcomings, we introduced two therapeutic agents with complementary mechanisms. Minocycline hydrochloride (MC) is a semi-synthetic tetracycline antibiotic with broad-spectrum antimicrobial, anti-inflammatory, and potent antioxidant activities (Zhang et al., 2019; Kim and Suh, 2009; Garrido-Mesa et al., 2013). Curcumin (Cur), a natural polyphenol from turmeric, is known for its potent anti-inflammatory, antioxidant, pro-angiogenic, and healing-promoting effects, including the stimulation of cell proliferation and migration (Kong et al., 2023; Patel et al., 2019; Fereydouni et al., 2018; Pulido-Moran et al., 2016). Despite its promising bioactivity, the clinical utility of Cur has been hindered by poor aqueous solubility and low bioavailability.

**Scheme 1:**
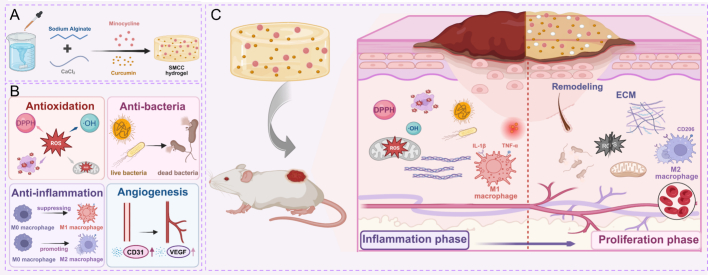
Schematic illustration of the synthesis of SMCC hydrogel with multifunctional properties for infected wound healing and skin reconstruction.

By co-loading MC and Cur into a Ca^2+^ crosslinked SA hydrogel, named SMCC, we developed a multifunctional system, which not only enhances the bioavailability of Cur for controlled local release but also harnesses the antibacterial, anti-inflammatory, and antioxidant effects of both drugs (Scheme 1). This integrated strategy provides a comprehensive platform to modulate the hostile wound microenvironment and promote the healing of chronic wounds.

## 2 Materials and methods

Materials and methods are available as supplementary materials.

## 3 Results and discussion

### 3.1 Fabrication and characterization of hydrogels

**Fig. 1:**
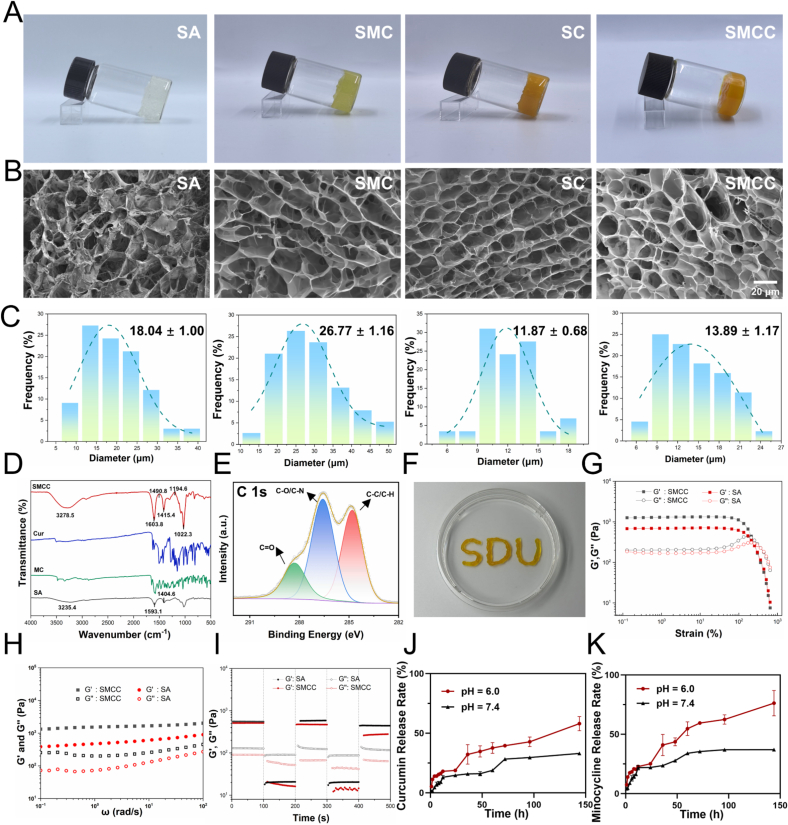
Characterization of hydrogels. A) Optical photographs of hydrogels. B) SEM images of hydrogels. C) The pore size distributions of hydrogels. D) FT-IR spectra patterns of SA, MC, Cur, and SMCC hydrogel. E) High-resolution C 1 s spectra of SMCC hydrogel. F) The injectability of SMCC hydrogels. Rheological studies of SA and SMCC hydrogels: G) frequency sweep under 1% strain condition, H) strain sweep at a frequency of 10 rad/s, I) alternating strain sweep at low (1%) and high (500%) strains with a set frequency of 10% rad/s. J) Cur and K) MC release behaviors of SMCC hydrogel at different pH values.

Before the addition of Ca^2+^, the mixed solution of SA, MC, and Cur exhibited fluidity and did not form a gel. Upon addition of Ca^2+^, SA underwent gelation and lost its fluidity (Fig. 1A). The individual or combined addition of MC and Cur did not hinder hydrogel formation, and the gel color gradually deepened with the incorporation of each component, leading to the designations SMC, SC, and SMCC. SEM images (Fig. 1B) revealed a three-dimensional porous structure in the hydrogels. Notably, the introduction of MC significantly altered the morphology and structure (Fig. 1C): the average pore size of the SMC group was 26.77 ± 1.16 μm, which was markedly larger than that of the SA group (18.04 ± 1.00 μm), indicating that MC participated in the gel network formation. This phenomenon can be attributed to the unique chemical structure of MC: the deprotonated hydroxyl group carries a negative charge, coordinating with metal ions, while the protonated dimethylamino group carries a positive charge, which can interact electrostatically with SA molecules (Liu et al., 2024). Such interactions between MC and SA contribute to enhanced drug loading capacity and suppression of burst release. Notably, the addition of curcumin leads to a reduction in porosity. This may be attributed to esterification and the formation of multiple hydrogen bonds between the phenolic hydroxyl groups of Cur and the hydroxyl and carboxyl groups on the alginate chains, which collectively enhance the crosslinking density and structural compactness of the polymer network (Cirillo et al., 2023; Sable et al., 2024). Elemental mapping of the SMCC hydrogel (Fig. S2) showed uniform distribution of C, N, O, and Ca, suggesting homogeneous dispersion of MC, Cur, and Ca^2+^ within the gel.

The FTIR spectra of the synthesized hydrogels are shown in Fig. 1D. In the SA spectrum, the broad peak at 3235.4 cm^−1^ is attributed to the O—H stretching vibration, while the characteristic peaks at 1404.6 cm^−1^ and 1593.1 cm^−1^ correspond to the symmetric and asymmetric stretching vibrations of the deprotonated carboxyl group (-COO^-)^, respectively (Xie et al., 2022). In the Cur spectrum, the absorption band in the range of 1600—1700 cm^−1^ originates from the stretching vibration of C=O (Zamani et al., 2024). In the composite SMCC spectrum, the O—H peak of SA shifts to 3278.5 cm^−1^ with noticeable broadening and sharpening, suggesting enhanced hydrogen bonding interactions. Compared to the spectrum of SA, the symmetric and asymmetric stretching vibrations of -COO^−^ in the SMCC hydrogel are shifted to 1415.4 and 1603.8 cm^−1^, respectively (Liu et al., 2017). The peak observed at 1022.3 cm^−1^ corresponds to C—O—C stretching of the polysaccharide backbone (Govindaraju et al., 2019), confirming that the alginate network remains structurally intact after incorporation of the drugs. Moreover, the peaks corresponding to the C=C bond of the aromatic ring at 1490.8 cm^−1^ and the C—N bond at 1194.6 cm^−1^ demonstrate the successful loading of MC. These results collectively confirm the successful preparation of the SMCC hydrogel.

XPS was further employed to confirm the elemental composition of SMCC hydrogel (Fig. S3). As shown in Fig. 1E and S4, the high-resolution C 1 s spectrum revealed three characteristic peaks: the peak at 284.8 eV is assigned to C—C bonds, which constitute the main framework of the polysaccharide backbone; the peak at 286.58 eV corresponds to C—O bonds; the peak at 288.29 eV is attributed to enol-type C=O bonds, indicating successful grafting of Cur onto the alginate chain (Dey and Sreenivasan, 2014). In the O 1 s spectrum, the peak at 531.56 eV is ascribed to C—O bonds, while the strong signal at 532.68 eV belongs to C=O bonds. An additional peak observed at 533.47 eV corresponds to O—C=O groups. The combined area of C—O and C=O bonds exceeds 60%, reflecting a high concentration of oxygen-containing functional groups on the surface. The N 1 s spectrum displays three distinct peaks. The peak at 399.39 eV is attributable to C—N bonds, while the pronounced peak at 400.11 eV is assigned to N—H bonds. These results collectively confirm the successful synthesis of the SMCC hydrogel at the level of chemical states, consistent with its expected molecular structure.

Injectability represents a critical feature of hydrogels, allowing facile clinical administration and effective filling of irregular wound cavities. The SMCC hydrogel could be smoothly loaded into and extruded from a syringe (Figs. 1F, S5), forming the letters “SDU” on the surface of a culture dish. Furthermore, adhesion tests performed on a finger joint (Fig. S6) confirmed its robust adhesive property, which promotes stable integration with dynamic wound surfaces and dressings, thereby enabling conformal adaptation to various irregular wound beds. Shear tests (Fig. S7A) also revealed that the viscosity of the SMCC hydrogel decreased linearly with increasing shear rate, confirming favorable shear-thinning behavior. Strain sweep measurements identified the critical point for the solid–liquid transition of the hydrogel (Fig. 1G). The incorporation of MC and Cur increased the yield strain, indicating enhanced cross-linking density and stronger intermolecular interactions. Within a frequency range of 0.1–100 rad/s at 1% strain, the storage modulus (G′) consistently remained higher than the loss modulus (G″) across all hydrogel formulations (Fig. 1H). Compared with the SA hydrogel, the SMCC hydrogel showed significantly increased moduli, confirming that abundant coordination interactions contribute to improved structural stability and mechanical performance. Alternating strain sweep tests were further conducted to evaluate the hydrogel recoverability (Fig. 1I). When the strain reached 500%, G′ dropped sharply below G″, indicating breakdown of the hydrogel network. Upon restoration of 1% strain, both G′ and G″ rapidly returned to their initial values, demonstrating efficient network reconstruction after large deformation. Finally, temperature-dependent rheological tests showed that G′ remained greater than G″ over a temperature range of 20–60 °C (Fig. S7B), indicating maintained structural stability of the hydrogel network within this thermal window.

Appropriate swelling capacity is essential for hydrogels to maintain mechanical integrity, preserve a moist wound environment, and support the healing process (Fan et al., 2024). The SMCC hydrogel exhibited a higher swelling ratio at pH 6.0 than at pH 7.4, with both reaching equilibrium within 48 h (Fig. S8). This phenomenon may be attributed to the competitive binding of H^+^ to carboxyl groups, which disrupts the Ca^2+^-mediated cross-links, leading to a looser, more porous network structure. This structural change facilitates the diffusion and release of both Cur (Fig. 1J, S9A) and MC (Fig. 1K, S9B), thereby improving therapeutic outcomes in the acidic microenvironment of infected wounds.

### 3.2 *In vitro* cell viability of hydrogels

An ideal hydrogel dressing must exhibit good biosafety as a fundamental prerequisite. To determine the optimal loading concentration of Cur, different concentrations (1, 2, 4, and 8 mg/mL) were incorporated into the SMC hydrogel. The results (Fig. S10A, S10B) showed that a high concentration (8 mg/mL) of Cur inhibited the proliferation of Raw 264.7 cells after 1 and 3 days of co-culture (with viability rates of approximately 80% and 76%, respectively). Therefore, 4 mg/mL was selected as the loading concentration for Cur.

**Fig. 2:**
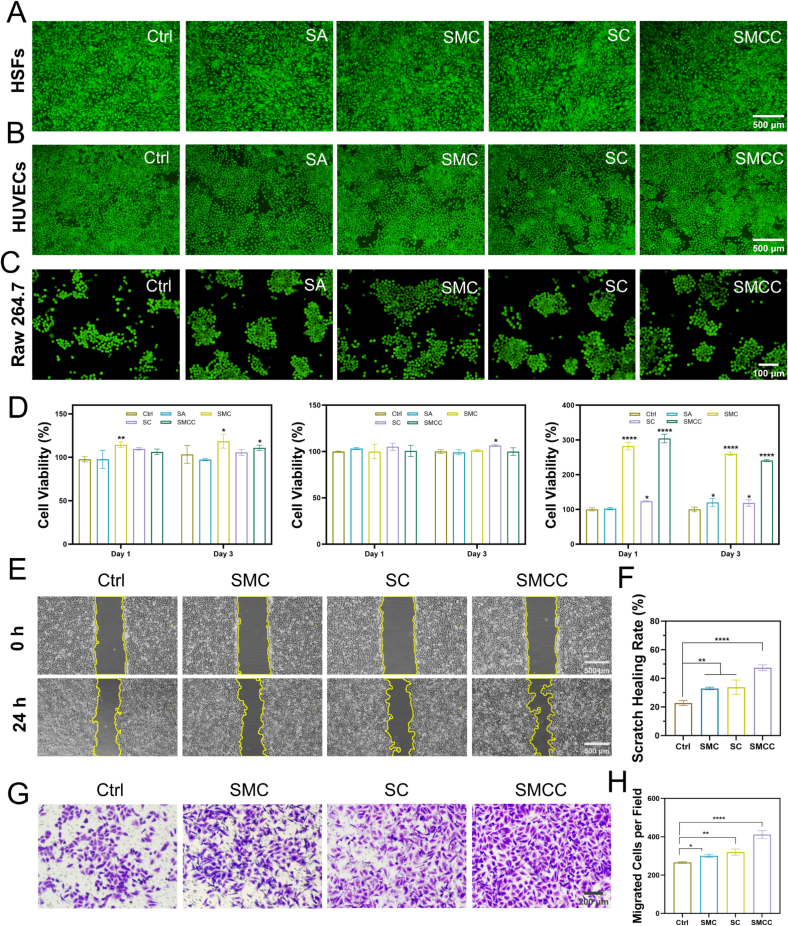
Cytocompatibility of hydrogels. Live/dead staining of A) HSFs, B) HUVECs, and C) Raw 264.7 after 3 days under various treatments. D) Cell viability experiment of HSFs, HUVECs, and RAW 264.7 after 1 and 3 days after treatment (n = 3). E) Scratch assay images of HUVECs after 24 h and F) statistics analysis (n = 3). G) Transwell experiment of HUVECs after 24 h and H) statistical analysis (n = 3).

In this study, the cytocompatibility of the SMCC hydrogel with HSFs, HUVECs, and RAW 264.7 cells was evaluated using the CCK-8 assay and live/dead staining. In the live/dead staining assay, live and dead cells were labeled with green and red fluorescence, respectively. The results (Fig. 2A–C) revealed that the majority of cells maintained normal morphology and displayed strong green fluorescence, with only sporadic red fluorescence from dead cells, indicating excellent cytocompatibility across all hydrogel groups.

Furthermore, cytocompatibility was assessed using the CCK-8 assay at 24 and 72 h after cell seeding. As shown in Fig. 2D, all hydrogel groups exhibited good cytocompatibility. Notably, compared to the control and SA groups, the SMC and SMCC hydrogels significantly promoted cell proliferation, particularly in RAW 264.7 cells and HSFs. In contrast, no significant differences were observed between the control and experimental groups for HUVECs.

### 3.3 Wound closing assays

**Fig. 3:**
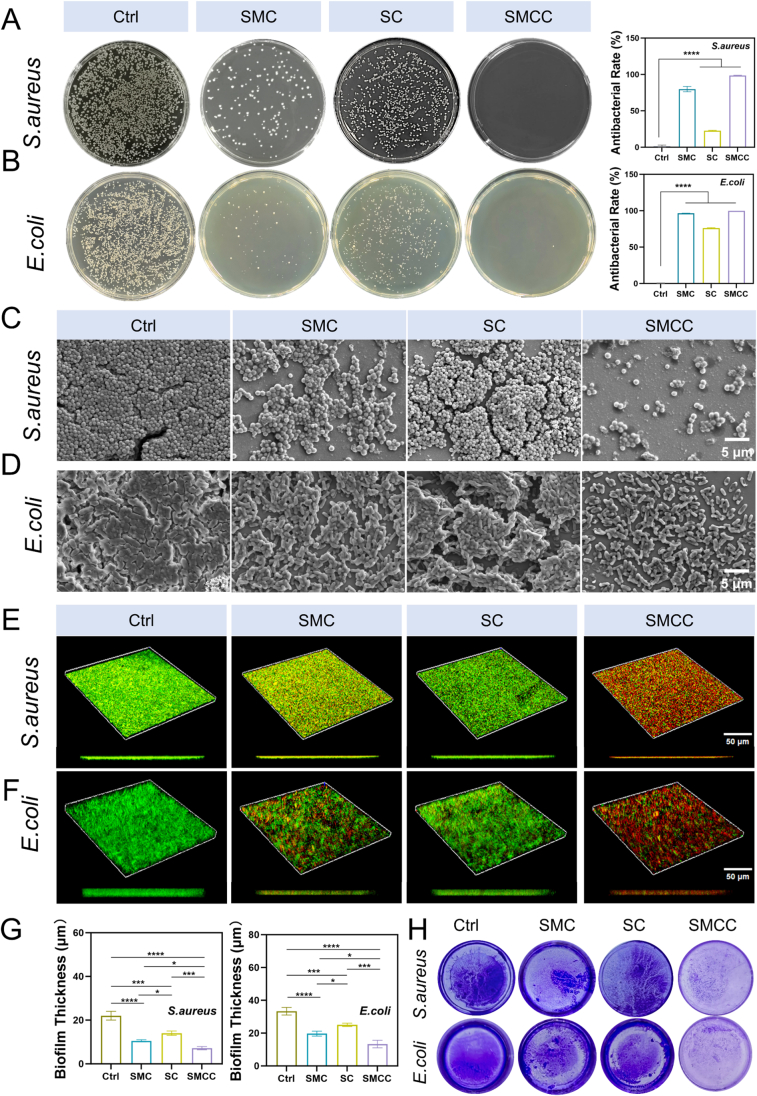
Antibacterial properties of hydrogels. A) Optical photographs of *S. aureus* and statistical analysis of the antibacterial ratio of hydrogels after treatments (n = 3). B) Optical photographs of *E. coli* colonies and statistical analysis of the antibacterial ratio of hydrogels after treatment (n = 3). SEM images of C) *S. aureus* and D) *E. coli* biofilms after treatment. Photographs of fluorescence staining of E) *S. aureus* and F) *E. coli* biofilms after treatment. G) Quantified average thickness of biofilms (n = 3). H) Photographs of crystal violet staining of biofilms after treatment.

Given that extracellular matrix remodeling is closely associated with cell proliferation and migration, we further evaluated the ability of the hydrogels to promote cell migration using scratch wound and Transwell assays. Scratch assay results (Fig. 2E) demonstrated that the SMCC group facilitated faster cell migration compared to the control, achieving approximately 47.4% wound closure after 24 h (Fig. 2F), suggesting that both MC and Cur contribute to enhanced cell motility. Consistently, the Transwell assay revealed a higher number of migrated HUVECs in the SMCC hydrogel-treated group (Fig. 3G, H). Together, these results underscore the potential of the SMCC hydrogel in supporting extracellular matrix remodeling and promoting epithelial regeneration.

### 3.4 *In vitro* antibacterial activity

To assess the antibacterial efficacy of the SMCC hydrogel, agar plate assays were performed. As shown in Fig. 3A and B, the colony counts of *S. aureus* and *E. coli* after 12 h of co-incubation with SMC, SC, and SMCC hydrogels were significantly lower than those in the control group. Quantitative analysis revealed inhibition rates of approximately 79.9%, 22.8%, 98.6%, 96.5%, 76.4%, and 99.9%, respectively, with statistically significant differences between each treatment group and the control (*p* < 0.01). Notably, the SMCC hydrogel exhibited markedly stronger antibacterial activity compared to the SMC and SC groups, suggesting a synergistic effect between Cur and MC.

Given that bacterial biofilms represent a major obstacle in chronic wound healing by sustaining local inflammation and impairing tissue repair (Ciofu et al., 2022), we further examined the anti-biofilm activity of the SMCC hydrogel. SEM images clearly showed that both *S. aureus* (Fig. 3C) and *E. coli* (Fig. 3D) in the control group developed mature, dense biofilms composed of closely packed cells. While the SMC and SC groups showed only limited biofilm inhibition, the SMCC treatment resulted in the thinnest and most fragmented biofilm structures. Moreover, compared to the intact and plump morphology of bacteria in the control group, *S. aureus* treated with SMCC displayed deformed and fused cell membranes (Fig. S11A), whereas *E. coli* exhibited collapsed and wrinkled surfaces (Fig. S11B).

Live/dead staining of biofilms (Fig. 3E, F) revealed green fluorescence corresponding to viable bacteria and red fluorescence indicating dead cells. In the control groups, biofilms of *S. aureus* and *E. coli* exhibited extensive green fluorescence with thicknesses of approximately 22 μm and 33 μm, respectively (Fig. 3G). Upon treatment with the different hydrogel formulations, varying intensities of red fluorescence appeared. The SMCC group displayed the most pronounced red fluorescence along with the thinnest biofilms, measuring about 7 μm for *S. aureus* and 13 μm for *E. coli*, confirming its potent antibacterial effect. Consistent outcomes were obtained from crystal violet staining assays (Fig. 3H). Quantitative analysis (Fig. S12) further indicated that the SMCC hydrogel achieved biofilm inhibition rates of approximately 79% against *S. aureus* and 60% against *E. coli*. Collectively, these results underscore the excellent antibacterial performance of the SMCC hydrogel.

The outstanding antibacterial activity of the SMCC hydrogel likely stems from the synergistic action of its components. MC contributes significantly through its burst release, primarily by binding to the 30S ribosomal subunit and inhibiting bacterial protein synthesis (Garrido-Mesa et al., 2013). It also possesses broad-spectrum antibacterial properties and can suppress biofilm formation (Fan et al., 2024). Concurrently, Cur enhances the overall efficacy, potentially by inhibiting bacterial virulence factors, impeding biofilm development, preventing bacterial adhesion, and interfering with the SOS response to DNA damage (Zheng et al., 2020).

### 3.5 Antioxidant and mitochondrial protective functions of hydrogels *in vitro*

**Fig. 4:**
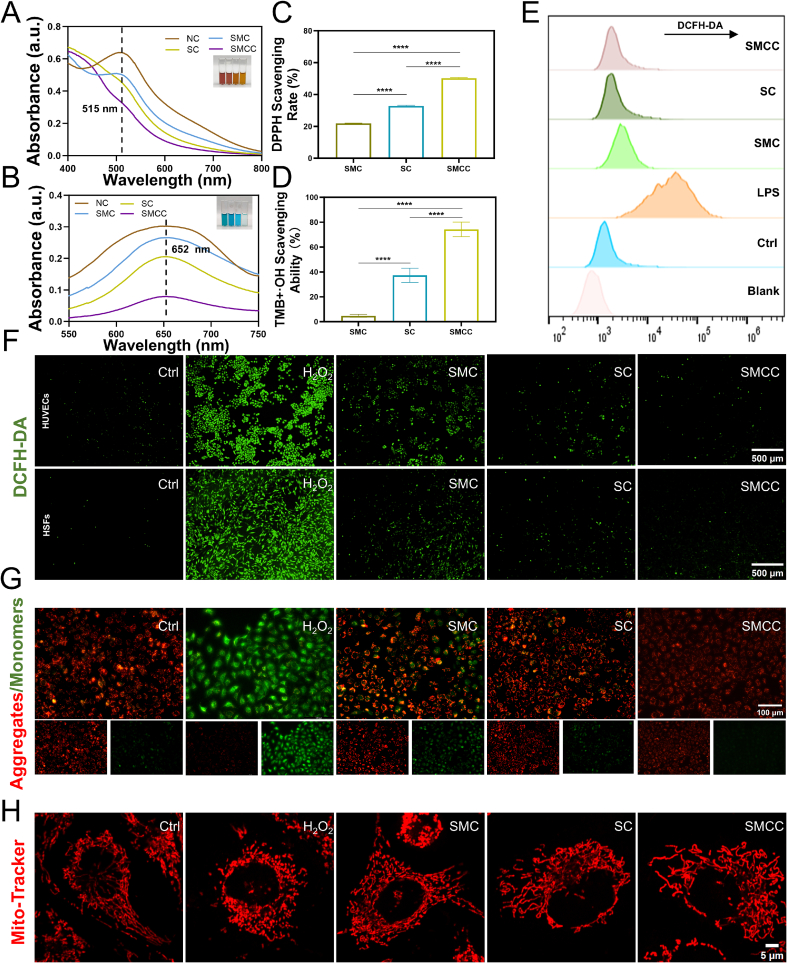
Antioxidant behavior of hydrogels. Scavenging of A) DPPH free radicals and B) ·OH by hydrogels. Statistical analysis of C) DPPH and D) ·OH scavenging. E) Flow cytometric analysis and F) representative images of DCFH-DA staining for intracellular ROS. G) Mitochondrial membrane potential levels measured with the JC-1 probe in HUVECs after treatment. H) Representative images of mitochondrial morphology of HUVECs.

The balance between oxidation and antioxidant defense is crucial for wound healing. Potent antioxidant activity helps reduce excess ROS, thereby alleviating oxidative stress-induced damage to cells. This benefits not only the initial inflammatory phase but also subsequent proliferation and remodeling stages (Wang et al., 2025). Accordingly, we evaluated the ability of the SMCC hydrogel to scavenge DPPH and hydroxyl radicals. As shown in Fig. 4A and B, a pronounced decrease in absorption peaks was observed, accompanied by a visible color change from dark to light, indicating a progressively enhanced ROS-scavenging capacity. Quantitative analysis (Fig. 4C, D) further confirmed that the SMCC hydrogel group exhibited the strongest antioxidant performance, with DPPH and ·OH scavenging rates reaching 50.2% and 74.2%, respectively. This effect can be attributed to the catechol groups in Cur, which are readily oxidized to quinone compounds and possess strong radical-trapping ability, thereby endowing the hydrogel with robust antioxidant properties (Kumari et al., 2022). Moreover, MC has been shown to directly scavenge ROS and neutralize them before they cause biological damage (Garrido-Mesa et al., 2013).

In infected wounds, excessive oxidative stress generates large amounts of free radicals, causing severe damage to cells and tissues that ultimately impedes wound healing (Wang et al., 2025). To systematically evaluate the protective effect of the SMCC hydrogel against oxidative stress, we established a cellular oxidative stress model using H_2_O_2_ and employed DCFH-DA as a fluorescent probe. Flow cytometry results (Fig. 4E, S13A) showed a significant leftward shift in the fluorescence intensity peak in the SMCC group. Similar outcomes were observed under microscopy: both HSFs and HUVECs treated with H_2_O_2_ alone exhibited intense fluorescence, whereas cells co-treated with H_2_O_2_ and SMC hydrogel, SC hydrogel, or SMCC hydrogel showed markedly weaker fluorescence signals (Figs. 4F, S13B). As expected, cells cultured with the SMCC hydrogel showed the lowest intracellular ROS levels among all groups, collectively demonstrating its excellent intracellular antioxidant activity.

Previous studies indicate that the phenolic hydroxyl and diketone functional groups in Cur can eliminate excess ROS, significantly enhance the viability of HUVECs, promote collagen deposition under oxidative microenvironments, and effectively facilitate wound healing (Phan et al., 2001; Panchatcharam et al., 2006). ROS are natural byproducts of oxygen metabolism. Under oxidative stress, excessive accumulation of ROS within cells leads to DNA damage, impairs cellular function, and hinders wound healing (Chen et al., 2025). Mitochondria, as metabolic centers of the cell, are particularly vulnerable to such oxidative damage. Therefore, restoring mitochondrial homeostasis is essential for reversing oxidative stress-related injury.

Therefore, we conducted a series of experiments to assess the protective effects of the SMCC hydrogel on mitochondrial function. Changes in mitochondrial membrane potential were evaluated using JC-1 staining (Fig. 4G, S13C). H_2_O_2_ treatment led to a significant decrease in the potential, whereas SMCC treatment restored it. Moreover, compared to the normal tubular morphology, mitochondria in HUVECs under oxidative stress exhibited fragmentation and swelling (Fig. 4H), characterized by shortened length and increased diameter (Fig. S14A, B). In contrast, hydrogel treatment resulted in increased mitochondrial length, indicating recovery of mitochondrial function. In summary, these results suggest that SMCC hydrogel alleviates H_2_O_2_-induced ROS overproduction and mitochondrial morphological damage. Interestingly, the antioxidant effect of the SMCC group was higher than that of the SMC and SC groups, which appears to be attributable to the synergistic antioxidant activity between MC and Cur.

### 3.6 Effect of hydrogels on angiogenesis *in vitro*

Angiogenesis plays a central role in wound healing, serving critical functions by establishing new blood vessels that deliver oxygen, nutrients, and growth factors to the wound site, remove metabolic waste, and regulate inflammation, proliferation, and tissue remodeling. These processes directly determine the speed and quality of wound repair (Sun et al., 2024). In chronic wounds, impaired vascularization is a major factor hindering healing, underscoring the need for hydrogels that can effectively deliver pro-angiogenic signals through targeted strategies.

**Fig. 5:**
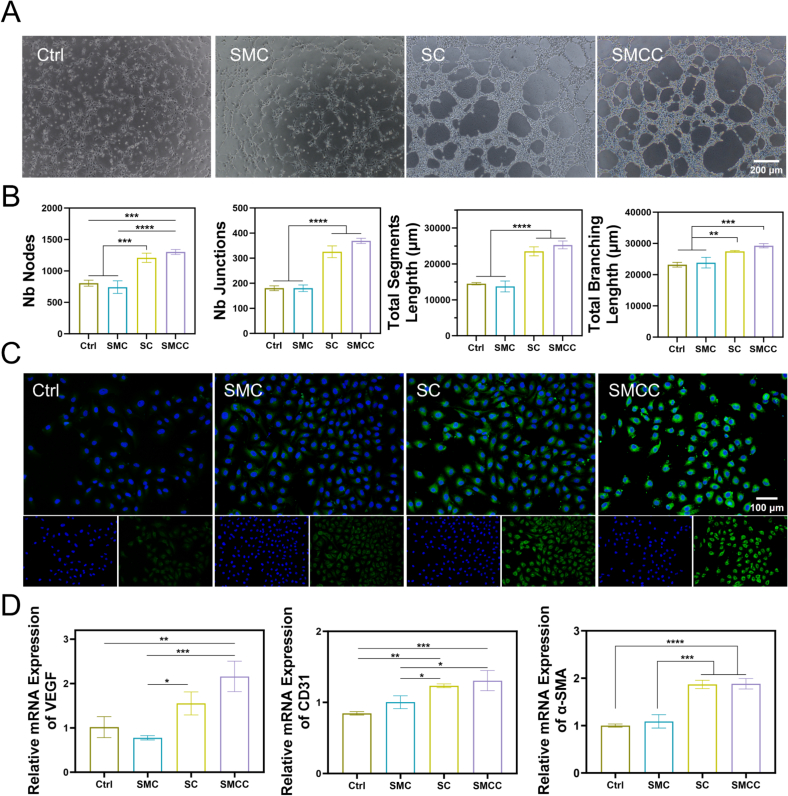
Angiogenesis evaluation of hydrogels. A) Typical images of tube formation of HUVECs incubated with different hydrogels for 8 h (n = 3). B) Quantitative analysis of the number of nodes, number of junctions, total segment length, and total branching length. C) Representative VEGF staining images among groups. D) Expression of VEGF, CD31, and α-SMA in HUVECs assessed by RT-qPCR (n = 3).

To evaluate the angiogenic potential of the developed hydrogels, HUVECs were treated with PBS (as the control group), SMC, SC, and SMCC hydrogels for 6 h, followed by assessment of vascular network formation. As shown in Fig. 5A, both the SC and SMCC hydrogel co-culture groups formed more extensive endothelial tubular structures within 6 h, compared to the control group. Notably, the SMCC hydrogel functioned as a potent pro-angiogenic agent, facilitating the formation of mature blood vessels and vascular networks within this timeframe. Key quantitative parameters for angiogenesis, including node number, junction number, total branch length, and total segment length, were evaluated. The results confirmed that the SMCC hydrogel significantly enhanced tube formation, with marked increases in all measured parameters relative to the control (Fig. 5B). These findings indicate that the SMCC hydrogel effectively stimulates angiogenesis in HUVECs, an effect likely attributable to the remarkable pro-angiogenic properties of Cur (Fereydouni et al., 2018).

Consistent with these observations, VEGF fluorescence signal intensity exhibited a similar trend in the hydrogel-treated groups (Figs. 5C, S15). To further investigate the molecular mechanisms underlying these effects, the expression of angiogenesis-related factors was analyzed *via* RT-qPCR. As demonstrated in Fig. 5D, the SMCC group significantly upregulated gene expression levels of vascular endothelial growth factor (VEGF), platelet endothelial cell adhesion molecule-1 (CD31), and alpha-smooth muscle actin (α-SMA) in HUVECs. Collectively, these results demonstrate that the SMCC hydrogel exhibits strong pro-vascularization capabilities *in vitro*, effectively promoting endothelial tube formation and modulating the expression of key vascular regulators. These properties highlight its potential as a promising strategy to enhance vascularization and support wound healing.

### 3.7 *In vitro* immune regulation of hydrogels

During wound healing, macrophages undergo phenotypic polarization, primarily into M1 and M2 subtypes from M0. These polarized macrophages play distinct and critical roles across various healing stages by regulating inflammatory responses, angiogenesis, cell proliferation, and ECM remodeling. The balance between M1 and M2 polarization directly influences the progression and quality of healing. M1 macrophages dominate the early inflammatory phase, serving as a primary defense against injury and infection. As inflammation subsides, macrophages polarize toward the M2 phenotype, becoming central regulators during the proliferation and remodeling phases, where they promote tissue regeneration, angiogenesis, and orderly ECM deposition (Rodrigues et al., 2019). Therefore, the ability of hydrogels as wound dressings to suppress M1 polarization is crucial for effective wound healing, as well as facilitating the transition from M0 to M2 phenotype.

**Fig. 6:**
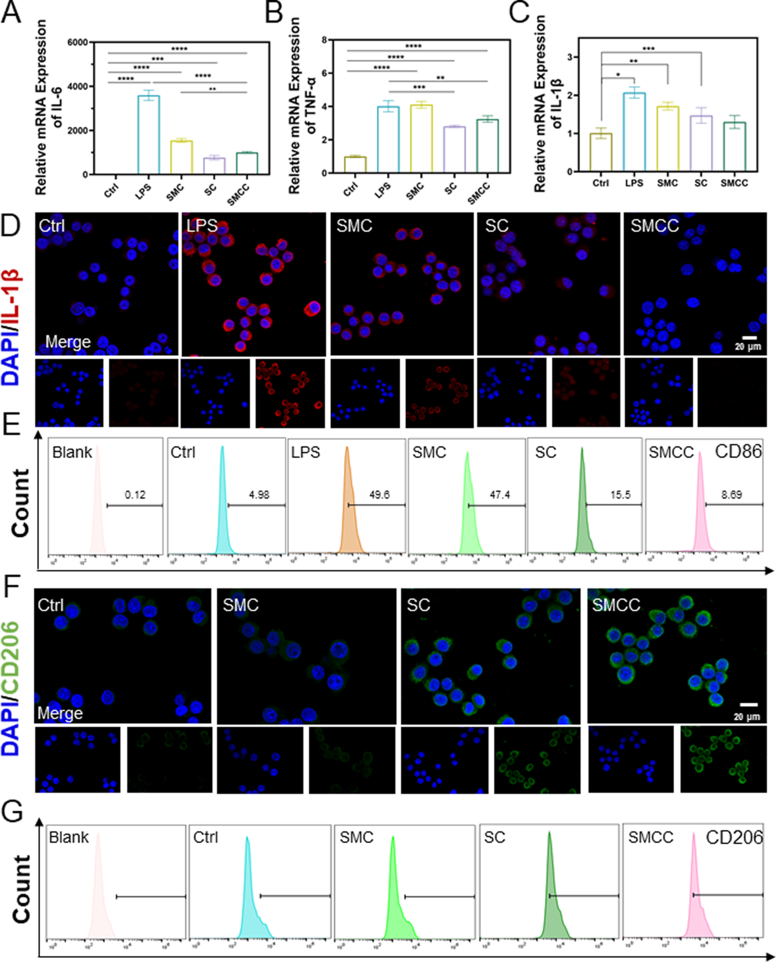
Anti-inflammation and *in vitro* immune regulation of hydrogels. Gene expression of A) IL-6, B)TNF-α, and C) IL-1β in macrophages assessed by RT-qPCR (n = 3). D) Representative fluorescence images of IL-1β. E) Macrophages polarization of CD86^+^ after treatment. F) Representative fluorescence images of CD206^+^. G) Macrophage polarization of CD206^+^ after treatment.

To investigate the immunomodulatory effects of the SMCC hydrogel, we first established an *in vitro* inflammation model by stimulating RAW 264.7 macrophages with lipopolysaccharide (LPS) and performed RT-qPCR analysis. Results showed that SMCC hydrogel treatment significantly downregulated the expression levels of inflammatory cytokines IL-6 (Fig. 6A), TNF-α (Fig. 6B), and IL-1β (Fig. 6C) while upregulating IL-10 (Fig. S16A) and CD206 (Fig. S16B) expression, demonstrating its pronounced anti-inflammatory effect in an inflammatory environment. The impact of the SMCC hydrogel on macrophage polarization was further assessed *via* immunofluorescence staining for the inflammatory cytokines IL-1β and the M2 marker CD206. Results indicated that LPS-treated cells exhibited high IL-1β (Fig. 6D, S17A) fluorescence intensity. In contrast, the SMC, SC, and SMCC hydrogel groups showed gradually reduced IL-1β fluorescence and progressively increased CD206 fluorescence compared to the LPS group and control group, respectively (Fig. 6F, S17B).

To further evaluate macrophage polarization in response to the hydrogels, flow cytometry was employed to analyze M1 marker CD86 (Fig. 6E) and M2 marker CD206 (Fig. 6G). The results showed that macrophages polarized toward the M1 phenotype under LPS stimulation, while CD86 expression was significantly reduced in the SMCC hydrogel group. Under unstimulated conditions, CD206 expression was low but markedly increased in the SMCC group. These findings indicate that the SMCC hydrogel effectively suppresses M1 polarization and promotes M2 polarization in macrophages. This effect may be attributed to the anti-inflammatory properties of the active components in the hydrogel: minocycline downregulates the mRNA expression of inflammation-related genes, including IL-6, IL-1β, and IL-2 (Garrido-Mesa et al., 2013). Meanwhile, curcumin alleviated intracellular oxidative stress and inflammatory responses, further inhibiting M1 polarization and enhancing M2 polarization.

### 3.8 *In vivo* infectious wound healing experiments

All animal procedures complied with the ARRIVE guidelines and received ethical approval from the Ethics Committee of the School of Stomatology, Shandong University (Approval No. 202503056), in accordance with the EU Directive 2010/63/EU on animal welfare.

**Fig. 7:**
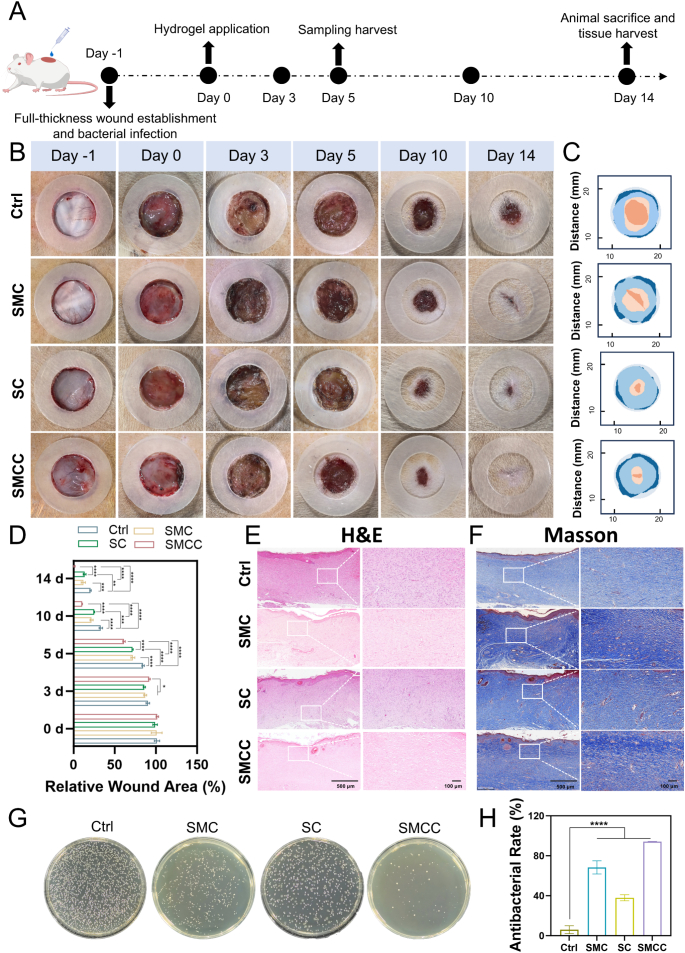
*In vivo* wound healing assessment. A) Schematic of the wound treatment procedure. B) Photographs of the wound site at different time points. C) Wound traces of the healing process. D) Quantification of relative wound area at different time points (n = 6). E) H&E and F) Masson's trichrome staining of wound tissue with different treatments on day 14. G) Representative plates of bacterial colonies from wound sites after different treatments on day 7 and H) statistical analysis (n = 3).

Based on the aforementioned experimental results, the SMCC hydrogel, characterized by favorable mechanical properties, sustained antibacterial activity, cytocompatibility, promotion of cell migration, mitigation of oxidative stress, and pro-angiogenic attributes *in vitro*, was anticipated to exhibit significant potential in promoting infected wound healing. As illustrated in Fig. 7A, full-thickness skin defects (1 cm in diameter) were created on the dorsum of Sprague-Dawley rats (male, 6 to 8 weeks, 200–250 g). Representative photographic images of the wound sites were captured on days 0, 3, 5, 10, and 14 (Fig. 7B, C). Wound contraction was quantified by comparing the wound area at each time point with the initial wound area on day 0 (Fig. 7D). On day 5, ulceration and inflammatory exudate persisted visible in all groups except the SMCC group. By day 10, the wound area in the SMCC group was significantly smaller than that in other groups, with a relative wound area of approximately 10.6%. After 14 days of treatment, all experimental groups showed reduced wound areas compared to the control group, with the SMCC group exhibiting the highest wound closure rate, indicating the most rapid healing progression.

To evaluate the effects of the SMCC sample on inflammatory response and angiogenesis during wound healing, skin tissues were collected on day 14 for H&E staining and analysis (Fig. 7E). The control group exhibited significant epidermal damage accompanied by varying degrees of inflammatory cell infiltration. In contrast, the SMCC group demonstrated a marked healing trend, characterized by newly formed epidermis, organized tissue structure, and significantly increased neovascularization and hair follicle regeneration. The newly formed blood vessels are essential for wound repair as they supply oxygen and nutrients to the damaged tissue microenvironment. Simultaneously, Masson's trichrome staining revealed that the SMCC hydrogel facilitated enhanced collagen formation and deposition. (Fig. 7F, S18). These results confirmed that the SMCC hydrogel effectively inhibited bacterial growth and promoted wound healing *in vivo*.

Cur contributes to wound healing through multiple aspects, with the function of shortening the inflammatory phase, promoting cell proliferation and tissue remodeling, and exerting antioxidant effects in the inflammatory milieu by scavenging reactive oxygen species and inducing the production of antioxidant enzymes. Additionally, Cur enhances fibroblast migration and proliferation, improves collagen deposition, and drives re-epithelialization processes during wound healing and tissue remodeling. Cur treatment has been shown to reduce healing time, improve collagen deposition, and increase fibroblast and vascular density in wounds, thereby enhancing impaired wound healing. Studies further indicate that Cur acts as a pro-angiogenic agent by inducing TGF-β, further promoting healing in both normal and impaired wound contexts (Joe et al., 2004).

To assess the *in vivo* antibacterial efficacy of the hydrogels, tissue samples were harvested from the wound sites on day 7 and subjected to colony counting. The number of residual *S. aureus* CFUs in the SMCC group was significantly lower than that in the control group (Fig. 7G, H). These results demonstrate the superior antibacterial activity of SMCC, attributable to the enhanced bactericidal effects of MC and Cur. Thus, the synergistic combination successfully improves anti-infective efficacy in treating *S. aureus*-infected skin wounds.

Finally, histological examination of the heart, liver, spleen, lungs, and kidneys from rats in all experimental groups revealed no significant inflammatory or pathological changes (Fig. S19), indicating the favorable biosafety of SMCC hydrogel. The *in vitro* hemolysis assay further supported its biocompatibility. As shown in Fig. S20, after interaction with blood and centrifugation, the negative control group displayed a clear supernatant above intact erythrocytes, while the positive control group showed a red supernatant due to hemolysis. All hydrogel groups exhibited clear, colorless supernatants similar to the PBS group. The hemolysis rates for SMC, SC, and SMCC were 1.7%, 0.6%, and 1.0%, respectively, all below the 5% safety threshold, confirming no hemolytic activity (Zhang et al., 2023).

### 3.9 Histology and immunofluorescence staining analysis

**Fig. 8:**
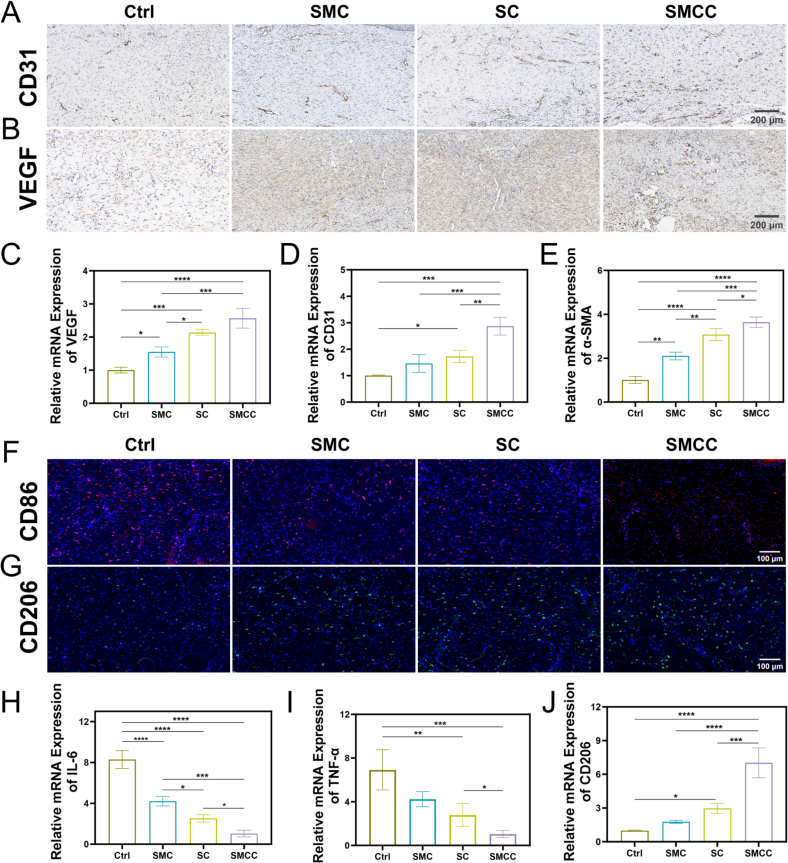
*In vivo* immunoregulatory and angiogenic capacities of hydrogels. Immunohistochemistry staining of A) CD31 and B) VEGF images of infected wound tissue in each group on day 14. Gene expression levels of C) VEGF, D) CD31, and E) α-SMA mRNA in scar tissues. (n = 3) Fluorescence staining of F) CD86 and G) CD206 images of infected wound tissue in each group on day 14. Gene expression levels of H) IL-6, I) TNF-α, and J) CD206 mRNA in scar tissues. (n = 3).

Accelerating angiogenesis is vital for tissue repair, as newly formed blood vessels provide immune surveillance, deliver oxygen and nutrients, and remove waste products during tissue reconstruction. To evaluate the angiogenic effect of hydrogels, immunohistochemical staining was performed for CD31 and VEGF in the regenerated tissue. As shown in Fig. 8A and B, while all groups expressed CD31 and VEGF, the SMCC group exhibited a marked increase in expression, suggesting higher vascular density. Quantitative analysis revealed that the fluorescence intensities of CD31 and VEGF in the treatment groups were significantly higher in all treatment groups compared to the control group, with the SMCC group showing the most pronounced enhancement (Fig. S21A, S21B). These results collectively demonstrate the excellent pro-angiogenic capacity of the SMCC hydrogel. Consistent with these findings, the RT-qPCR analysis revealed similar trends in the expression of angiogenesis-related factors of wound tissues (Fig. 8C–E).

Wound healing is a highly coordinated physiological process (Joe et al., 2004). Persistent inflammation can disrupt this progression and impede the transition from the inflammatory to the proliferative phase, thereby inhibiting polarization to the M1 phenotype and promoting the M2 phenotype is crucial for resolving inflammation and advancing healing. Therefore, immunofluorescence staining was conducted for the M1 marker CD86 and the M2 marker CD206 to further evaluate the hydrogel's impact on the inflammatory microenvironment. Compared with the control group, the SMCC group showed attenuated CD86 signaling (Fig. 8F) and enhanced CD206 expression around the wound (Fig. 8G), indicating a reduction in M1 macrophages and an increase in M2 macrophages. Above was further supported by RT-qPCR (Fig. 8H–J), which showed lower expression levels of inflammatory cytokines (IL-6, TNF-α) and higher expression levels of M2 marker (CD206) of wound tissues. Together with prior *in vitro* findings, these data demonstrate that SMCC hydrogel alleviates inflammation by promoting macrophage polarization toward the M2 subtype with the excellent ability to modulate the wound microenvironment and accelerate wound healing.

## 4 Conclusion

Our study successfully developed and systematically evaluated a pH-responsive multifunctional hydrogel dressing, termed SMCC. Crosslinked *via* dynamic Schiff base bonds and metal coordination, the hydrogel exhibits accelerated drug release at wound conditions, along with excellent injectability, adaptability, and self-healing capacity, allowing it to conform perfectly to irregular wounds. *In vitro* experiments demonstrated that the SMCC hydrogel possesses remarkable antibacterial, antioxidant, and anti-inflammatory properties. Its antibacterial effects stem from the synergistic action of MC and Cur, which disrupt bacterial membrane integrity and inhibit biofilm formation. The hydrogel significantly scavenges excess ROS, alleviates oxidative stress, and protects cells from damage. More importantly, SMCC markedly enhanced the tube-forming ability of HUVECs and modulated macrophage polarization by suppressing inflammatory cytokines and M1 phenotype while promoting the anti-inflammatory and reparative M2 phenotype, thereby remodeling the inflammatory microenvironment. *In vivo* studies using a rat full-thickness infected skin defect model further confirmed the therapeutic efficacy of the SMCC hydrogel. The SMCC-treated group exhibited the highest wound closure rate, the most robust tissue regeneration, and the strongest bacterial clearance capability. Histological analysis revealed significantly promoted collagen deposition, enhanced epithelial regeneration, and accelerated angiogenesis. In conclusion, the SMCC hydrogel represents an effective strategy for promoting wound healing and offers a highly promising approach for relevant clinical applications.

## CRediT authorship contribution statement

**Ziwei Wang:** Writing – original draft, Visualization, Project administration, Methodology, Investigation, Formal analysis, Data curation. **Hongxia Zhao:** Writing – review & editing, Visualization, Methodology, Formal analysis. **Longxuan Zhan:** Visualization, Methodology, Investigation. **Yifei Zhang:** Software, Methodology. **Yongzhe Liu:** Software, Methodology. **Xiaoni Ma:** Writing – review & editing, Supervision, Funding acquisition, Conceptualization. **Baojin Ma:** Writing – review & editing, Supervision, Resources, Funding acquisition, Conceptualization.

## Declaration of competing interest

The authors declare that they have no known competing financial interests or personal relationships that could have appeared to influence the work reported in this paper.

## Data Availability

Data will be made available on request.
