# Supplementary material for: pH-responsive injectable hydrogel dressing integrating antibacteria, antioxidation, anti-inflammation, and angiogenesis for infected wound healing
Source: Int J Pharm X. 2026 Jun 27;12:100591. doi: 10.1016/j.ijpx.2026.100591 (PMC13330663; doi:10.1016/j.ijpx.2026.100591)
Supplement: Supplementary file 1 — Supplementary material [file mmc1.docx]

Supporting Information

pH-Responsive Injectable Hydrogel Dressing Integrating Antibacteria, Antioxidation, Anti-inflammation, and Angiogenesis for Infected Wound Healing

Ziwei Wang, Hongxia Zhao, Longxuan Zhan, Yifei Zhang, Yongzhe Liu, Xiaoni Ma^*^, Baojin Ma^*^

**Address**: Department of Implantology & Tissue Engineering and Regeneration, School and Hospital of Stomatology, Shandong University & Shandong Key Laboratory of Oral Tissue Regeneration & Shandong Engineering Research Center of Dental Materials and Oral Tissue Regeneration & Shandong Provincial Clinical Research Center for Oral Diseases, Jinan, 250012, China

**E-mail**: baojinma@sdu.edu.cn; maxiaoni0@126.com

**1. Materials and methods**

**1.1 Materials**

Sodium alginate (CAS: 9005-38-3, MW: 20–50 kDa, M: G = 1:1, Viscosity: 15–60 mpa·s), calcium chloride (CaCl_2_·2H_2_O), and curcumin (CAS: 458–37-7, GR, MW: 368.38 g/mol) were purchased from Macklin (Shanghai, China). Minocycline hydrochloride (CAS:13614-98-7, GR, MW:493.94 g/mL) was purchased from Aladdin (Shanghai, China). 2,2-Diphenyl-1-picrylhydrazyl (DPPH, CAS: 1898-66-4, MW: 394.32, 97.00%), Crystal Violet Ammonium Oxalate Solution (G1062), Enhanced Mitochondrial Membrane Potential Assay Kit with JC-1 (M8650), Brain Heart Infusion Medium (LB0720), and Brain Heart Infusion Agar (LA0680) were obtained from Solarbio (Beijing, China). Cell Counting Kit8 (CCK8) was purchased from Biosharp (Anhui, China). Live/Dead Bacterial Staining Kit was purchased from Biosciences (USA). Live/dead staining kit (C1371S), Mito-Tracker Red CMXRos (C1049B), and Reactive Oxygen Species Assay Kit (S0035S) were purchased from Beyotime (Shanghai, China).

**1.2 Preparation of hydrogels**

Based on established methodologies[1], we prepared stock solutions by dissolving 20 mg of Cur in 5 mL of anhydrous ethanol and 25 mg of MC powder in 5 mL of deionized water, respectively. The MC solution (5 mg/mL) was introduced into deionized water under continuous magnetic stirring at 1000 rpm, followed by the addition of 200 mg of sodium alginate powder to form a homogeneous precursor solution. Subsequently, varying volumes of the Cur solution were incorporated, and cross-linking was initiated by dropwise addition of a 1% calcium chloride solution, resulting in the formation of a solid hydrogel. The gelation was confirmed by inverting the container for 1 minute without observing flow. Hydrogels prepared with MC or Cur alone were designated as SMC and SC, respectively.

**1.3 Structural characterization of hydrogels**

The cross-sectional morphologies of the freeze-dried hydrogels were examined using scanning electron microscopy (SEM, S-4800; Hitachi, Japan) to analyze pore structures, and elemental analysis was performed via the aforementioned SEM equipment. Fourier transform-infrared (FT-IR) spectroscopy was conducted within the spectral range of 4000–500 cm^-1^ to characterize the freeze-dried hydrogels as well as the powders of SA, Cur, and MC by a Thermo Nexus 670 spectrometer (Thermo Fisher, America). Additionally, X-ray photoelectron spectroscopy (XPS) was employed to investigate the elemental composition and cross-linking status of the target hydrogels through Thermo Scientific ESCALAB 250Xi+ (Thermo Fisher, America).

**1.4 Rheology performances of hydrogels**

The rheological properties of the hydrogels from each group were characterized using a rheometer (Anton Paar, MCR 302s, 135 Austria). Amplitude sweep tests were performed at a constant frequency of 10 rad/s to measure the storage modulus (G') and loss modulus (G''). Frequency sweep analyses were conducted on the target hydrogels under a fixed strain of 1%. Alternating strain sweep experiments were implemented by applying low (1%) and high (500%) strain conditions sequentially. Flow sweep test was studied at shear rates ranging from 0.1 to 10 s^−1^ to evaluate the shear-thinning behavior of hydrogels. The temperature-dependent behavior was investigated across a temperature range of 20 to 60°C.

**1.5 Injectability of hydrogels**

The SMCC hydrogel was aspirated into a 1 mL single-channel syringe and subsequently employed to write the characters “SDU” within a petri dish.

**1.6 pH-responsive swelling and release behaviors of hydrogels**

1 mL SMCC hydrogel was immersed in 10 mL of PBS at different pH values (pH = 6.0 and 7.4) and incubated at 37°C with shaking at 100 rpm. At predetermined time intervals (1h, 2h, 4h, 6h, …), 1 mL of the release medium was sampled and replaced with fresh PBS to maintain volume. The collected samples were stored at –20 °C in the dark until analysis. The cumulative release of Cur and MC was quantified using a microplate reader. The cumulative amount of drug released was calculated using the following equation:

$$\text{Q}_{\text{n}}\text{=}\text{C}_{\text{n}}\text{⋅}\text{V}_{\text{0}}\text{+}\text{v}\sum_{\text{i}\text{=1}}^{\text{n-1}} \text{C}_{i}$$

where C_n_ is the concentration measured in the n-th sample, V_0_ is the total volume, v represents the volume of each sample withdrawn, and C_i_ represents the concentrations measured in the previous samples (i=1, 2, …, n−1). The cumulative release percentage (P_n_) was then determined as:

$$\text{P}_{\text{n}}\text{=}\frac{\text{Q}_{\text{n}}}{\text{D}}\text{×100\%}$$

where D is the theoretical total drug content.

Simultaneously, the swelling ratio was evaluated under the same conditions using the wet gravimetric method by weighing the hydrogels at various time points.

**1.7 *In vitro* cell experiments**

**1.7.1 Cell culture**

Human umbilical vein endothelial cells (HUVECs) and human skin fibroblasts (HSFs) were obtained from the cell bank of the Type Culture Collection of the Chinese Academy of Sciences (Shanghai, China). The RAW 264.7 mouse leukemia cell line was purchased from Procell Life Science & Technology Co., Ltd. (Wuhan, China). HSFs and Raw 264.7 macrophages were maintained in high-glucose (4.5 g/L) Dulbecco’s modified Eagle medium (DMEM, Basal-media, China) supplemented with 10% Fetal Bovine Serum (FBS, Gibco) and 1% penicillin-streptomycin. HUVECs were cultured in an endothelial cell medium (ECM, Nexell, China) containing 1% endothelial cell growth supplement (ECGS, Nexell) and 5% Fetal Bovine Serum (FBS, Nexell). Cells were incubated in a 5% CO2 atmosphere at 37 °C.

**1.7.2 Biocompatibility assessment**

Hydrogel extracts were obtained by immersing sterilized hydrogel samples in PBS in a shaking table (70 rpm/min) at 37 °C for 24 hours and then stored at 4 °C in the dark for further experiments. To assess the biosafety of hydrogels, the CCK-8 assay and live/dead fluorescence staining were utilized. In the live/dead staining assay, HUVECs, HSFs, and RAW 264.7 macrophages were seeded at a density of 5 × 10^4^ cells per well in 12-well plates, cultured in complete medium at 37 °C in a 5% CO_2_ incubator for 24 hours. Then, hydrogel extracts and PBS were added to each well, followed by co-culturing for 3 days. The fluorescence signals were observed and captured using a fluorescence microscope (OLYMPUS IX73; Japan). As to the CCK-8 assay, after the incubation period, the culture medium was replaced with 100 μL of fresh medium containing 10% CCK-8 reagent. After incubating for 30 minutes in the dark, the absorbance was measured at 450 nm using a microplate reader (BioTek, Synergy H1, America).

**1.7.3 Cell migration assay**

Cell migration was evaluated using a scratch assay and a transwell migration assay. For the scratch assay, a confluent monolayer of HUVECs was scratched using a sterile 1 mL pipette tip and treated with serum-reduced medium containing hydrogel extracts. Images were captured at 0 h and 24 h using an inverted microscope (Olympus IX73, Japan). Quantitative analysis of the wound closure area was performed using ImageJ software.

For the Transwell migration assay, HUVECs were seeded into the upper chamber, with hydrogel extracts added to the lower chamber. Following incubation for 24h, the cells were fixed, washed, and stained with 0.1% crystal violet solution. The migrated cells were photographed by a microscope and quantified using ImageJ.

**1.8 *In vitro* antibacterial activity**

**1.8.1 Colony formation assay**

Antibacterial activity of the hydrogel against *S. aureus* and *E. coli* was evaluated using the colony counting method. Briefly, 1 mL of each hydrogel formulation was placed at the bottom of tubes, and 1 mL of bacterial suspension in LB broth (1.0 × 10^6^ CFU/mL) was added on top. The mixtures were co-incubated at 37 °C on a shaker at 100 rpm for 12 h. After incubation, the bacterial suspensions were serially diluted to appropriate concentrations, and 50 µL of the supernatant from each dilution was evenly spread onto agar plates. The plates were further incubated at 37 °C for 12 h. Digital photographs of the colonies were captured, and colony numbers were quantified using ImageJ software. For each hydrogel formulation, experiments were performed in triplicate, and the mean colony count was used to calculate the inhibition rate:

$$\text{Inhibition rate =(1-}\frac{N_{S}}{N_{C}}\text{)×100\%}$$

where is N_S_ and N_C_ represent the colony counts of the sample group and the control group, respectively.

**1.8.2 Biofilm inhibition**

Bacterial suspensions of *S. aureus* and *E. coli* at a concentration of 1.0 × 10^6^ CFU/mL in LB broth were incubated with hydrogel extracts from each experimental group, using an equal volume of physiological saline as the control group. The mixtures were incubated at 37 °C for 48 hours to obtain biofilm formation. The developed biofilms were fixed overnight with 4% paraformaldehyde, washed with PBS, and subjected to gradient dehydration. Following complete drying, the samples were sputter-coated with gold and observed using scanning electron microscopy (SEM) to evaluate biofilm morphology.

The anti-biofilm efficacy of the hydrogels was further assessed via live/dead bacterial staining. The stained biofilms were visualized using confocal laser scanning microscopy (LSM, ZEISS MIC-SYSTEM) to analyze biofilm architecture and thickness.

For quantitative analysis, the biofilms were stained with 1% crystal violet for 30 minutes, rinsed thoroughly with distilled water, and air-dried at room temperature before imaging. The retained dye was subsequently dissolved in absolute ethanol, transferred to a 96-well plate, and measured at an optical density of 590 nm using a microplate reader.

**1.9 Antioxidant and intracellular antioxidative tests of hydrogels.**

**1.9.1 Free radical scavenging assay**

The DPPH (2,2-diphenyl-1-picrylhydrazyl) radical scavenging assay was conducted to evaluate the *in vitro* antioxidant activity of the hydrogels. Specifically, a 1 mM DPPH stock solution was diluted with ethanol to obtain a 0.2 mM working solution. Then, 0.1 mL of each hydrogel sample (SMC, SC, SMCC) was placed at the bottom of a separate 5 mL EP tube, and 2 mL of the 0.2 mM DPPH solution was added. A control group containing only the DPPH solution without hydrogel was prepared for comparison. All samples were incubated at room temperature in the dark for 30 minutes. The color changes before and after incubation were visually observed and photographically recorded by a UV-spectrophotometer (SHIMADZU-UV-2600 I, Japan) at 515 nm to assess the radical scavenging capacity.

**1.9.2 ·OH scavenging capacity**

To evaluate the hydroxyl radical (·OH) scavenging capacity of the hydrogels, a spectrophotometric method utilizing 3,3′,5,5′-tetramethylbenzidine (TMB) as a colorimetric probe was employed. This assay detects residual ·OH generated via the Fenton reaction, which occurs between copper ions (Cu^2+^) and hydrogen peroxide (H_2_O_2_). Specifically, 100 μL aliquots of hydrogel samples from each experimental group were incubated with 10 mM H_2_O_2_ and 1 mM Cu^2+^ at room temperature to initiate radical generation. Subsequently, the mixtures were combined with TMB solution and incubated for 30 minutes to allow color development, during which TMB is oxidized by ·OH to produce a blue-colored product. The absorbance of the resulting solution was measured at 652 nm using a UV-spectrophotometer.

**1.9.3 Intracellular ROS scavenging capability of hydrogels**

Intracellular ROS levels were evaluated using the DCFH-DA probe. Briefly, we seeded HSFs and HUVECs at 5 × 10^4^ cells per well in 12-well plates and incubated at 37°C with 5% CO_2_ for 24 hours. Following treatment with 500 μM H_2_O_2_ and hydrogel extracts for 12 hours, H_2_O_2_ alone was used as the positive control, and an equal volume of PBS was used as the negative control. After incubation, the cells were stained with 10 μm DCFH-DA in DMEM at 37°C in the dark for 30 minutes. Following staining, the cells were washed three times with PBS to thoroughly remove excess probe. Finally, the cells were immersed in pure DMEM, and fluorescence was observed under a fluorescence microscope. Quantitative analysis was performed using ImageJ software. For flow cytometry analysis, the cells were collected into centrifuge tubes. The cells were washed twice with PBS, and then incubated with 500 μL of 10 μM DCFH-DA working solution at 37 °C in the dark for 20 min. Subsequently, the cells were centrifuged at 1000 rpm for 5 min, the staining solution was removed, and the cells were washed with PBS and resuspended. The fluorescence intensity was immediately measured using a flow cytometer (BD Biosciences, USA) with the FITC channel.

**1.9.4 Mitochondrial homeostasis regulation of hydrogels**

Cells were cultured following the aforementioned standardized protocol. Mitochondrial membrane potential was assessed using the fluorescent probe JC-1. The fluorescence intensities of JC-1 aggregates (red) and monomers (green) were visualized under a microscope and quantified using ImageJ. Mitochondrial morphology was examined by confocal microscopy (LSM, ZEISS MIC-SYSTEM) after staining with Mito-Tracker. The length and diameter of mitochondria were quantified using the Mitochondrial Analyzer plugin in ImageJ software.

**1.10 Matrigel assays *in vitro***

The angiogenic effect of the hydrogel was evaluated using an *in vitro* tube formation assay. HUVECs were treated with hydrogel extracts from each group for 48 hours, with a control group receiving no extract. 20 μL of Matrigel was added to a 24-well plate and incubated at 37 °C for 45 minutes to solidify. Then, HUVECs from each group were seeded at a density of 1.5 × 10^5^ cells per well onto the solidified Matrigel layer. Tube formation was observed and photographed under a microscope at 6 hours. Quantitative analysis was performed using ImageJ software.

**1.11 Cellular immunofluorescence staining**

Formaldehyde-fixed cells were incubated with primary antibodies specific for IL-1β (WLH3903), CD206 (WL06177), VEGF (WL00009b), and CD31 (WL03674), followed by incubation with appropriate fluorescently labeled secondary antibodies. Cell nuclei were counterstained with DAPI (4',6-diamidino-2-phenylindole), and the samples were imaged using a laser scanning confocal microscope (LSM, ZEISS MIC-SYSTEM). Quantitative analysis of relative fluorescence intensity was performed using ImageJ software.

**1.12 Real-time quantitative polymerase chain reaction (RT-qPCR)**

Total RNA was extracted from samples using Trizol reagent (AG, China), and its concentration and quality were verified using a NanoDrop 2000 spectrophotometer (Thermo Fisher Scientific, USA). Reverse transcription was then performed with the SPARK script II All-in-one RT SuperMix (Sparkjade, China) to synthesize cDNA. Amplification was carried out on a Bio-Rad CFX Duet system (USA) using 2 × SYBR Green qPCR Master Mix (Sparkjade, China). The primers and amplicons used for real-time quantitative reverse transcription polymerase chain reaction (RT-qPCR) analysis are listed in Supplementary Table 1.

**1.13 Flow cytometric analysis of macrophage phenotypes**

RAW 264.7 cells were seeded at a density of 3 × 10^5^ cells per well in 6-well plates and incubated for 24 hours at 37 °C under 5% CO_2_. The cells were then treated with 100 ng/mL lipopolysaccharide (LPS) along with hydrogel extracts from each experimental group for 24 hours. Subsequently, the cells were fixed with 4% paraformaldehyde and permeabilized with 0.1% Triton X-100. Immunostaining was performed by incubating the cells with fluorescently conjugated antibodies against CD86 and CD206 for 30 minutes at room temperature. Macrophage polarization was quantitatively evaluated using a flow cytometer, with data analysis focusing on the expression levels of M1 (CD86) and M2 (CD206) phenotypic markers.

**1.14 *In vivo* animal experiments**

**1.14.1 Animal model establishment and treatment protocols**

To evaluate the wound healing efficacy of the SMCC hydrogel, an experiment was conducted using 12 healthy male Sprague-Dawley rats aged 6–8 weeks (200–250 g, SPF). The rats were purchased from Vital River Laboratory Animal Technology Co., Ltd. (License No. SCXK (Zhe) 2024–0001). All animal procedures complied with the ARRIVE guidelines and received ethical approval from the Ethics Committee of the School of Stomatology, Shandong University (Approval No. 202503056), in accordance with the EU Directive 2010/63/EU on animal welfare.

The rats were anesthetized with isoflurane, and four full-thickness skin defects (diameter: 10 mm) were created on the dorsal region of each rat. The wounds were inoculated with Staphylococcus aureus suspension (100 μL, 1.0 × 10^8^ CFU/mL) to establish an infected wound model. The control group was treated with PBS, while the experimental groups received SM, SC, and SMCC hydrogel applications, respectively. Hydrogel dressings were replaced every two days throughout the study to maintain consistent therapeutic exposure. Wound healing progression was monitored and documented via macroscopic observation and photographic recording at days 0, 3, 5, 10, and 14 post-intervention.

**1.14.2 Histological and quantitative analysis of skin wounds**

Skin tissue samples were fixed in 4% paraformaldehyde, dehydrated through graded ethanol and xylene series, embedded in paraffin, and sectioned at 5 μm thickness for histological evaluation using H&E and Masson's staining. Subsequent imaging and analysis were performed via optical microscopy. For qPCR analysis, wound tissues from each group were homogenized in Trizol reagent, centrifuged to isolate supernatant, and subjected to gene expression analysis of IL-6, TNF-α, CD206, VEGF, CD31, and α-SMA using established protocols. Additionally, major organs (heart, liver, spleen, lungs, kidneys) were collected post-euthanasia and assessed for *in vivo* biosafety of hydrogels through H&E staining; wound healing rate was calculated as:

$$\text{W}\text{ound healing ratio = }\frac{\text{S}_{\text{0}}\text{ - }\text{S}_{\text{n}}}{\text{S}_{\text{0}}}\text{ × 100\%}$$

here S_0_ is the initial wound area on day 0, and Sn is the area of the unhealed wound on days 3,5, 10, and 14, respectively.

**1.15 Hemolytic tests**

Fresh blood was collected from the hearts of SD rats (male, 200–250 g) and centrifuged at 2000 rpm for 10 minutes at 4 °C. The resulting serum was discarded, and an equal volume of 0.9% saline was added to the red blood cell (RBC) pellet. This process was repeated three times to ensure complete removal of residual serum. Finally, the obtained RBC pellet was resuspended in fresh saline to prepare a 5% v/v RBC suspension.

A volume of 100 μL of hydrogel was mixed with 1 mL of the RBC suspension in a 1.5 mL anticoagulant tube. The mixture was incubated at 37 °C for 1 hour. A 0.1% Triton X-100 solution was used as the positive control, while saline served as the negative control. After incubation, the hydrogel was removed, and the mixture was centrifuged at 12,000 rpm for 5 minutes. Digital photographs were captured, and the absorbance of the supernatant at 540 nm was measured using a microplate reader. The hemolysis rate of the hydrogel was evaluated using the following formula:

$$\text{H}\text{emolysis ratio = }\frac{\text{O}\text{D}_{\text{H}} \text{- O}\text{D}_{\text{S}}}{\text{O}\text{D}_{\text{T}} \text{- O}\text{D}_{\text{S}}}\text{ × 100\%}$$

where OD_H_, OD_P_, and OD_S_ denote the OD values of the hydrogel, saline, and Triton-X100, respectively.

**1.16 Statistical analysis**

All data analyses were conducted using Origin software (Version 2024) and GraphPad Prism (Version 10.1.2). The data are represented as mean ± SD (n≥3). The data were analyzed by the one-way ANOVA test. Statistical significance was considered as **P* < 0.05, ***P* < 0.01, ****P* < 0.001, *****P* < 0.0001.


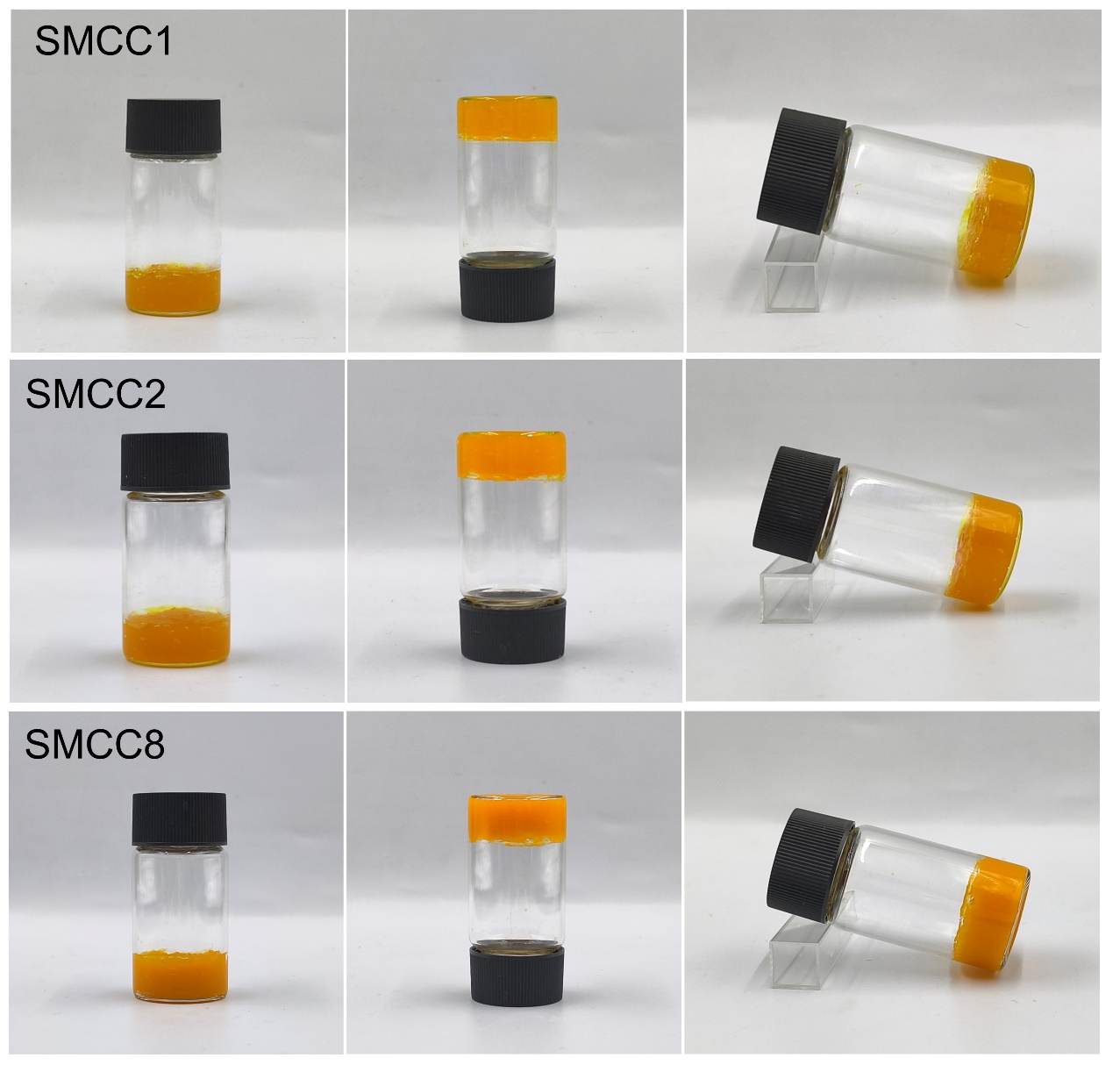


Figure S1. The gelation process of hydrogels with different Cur concentrations.


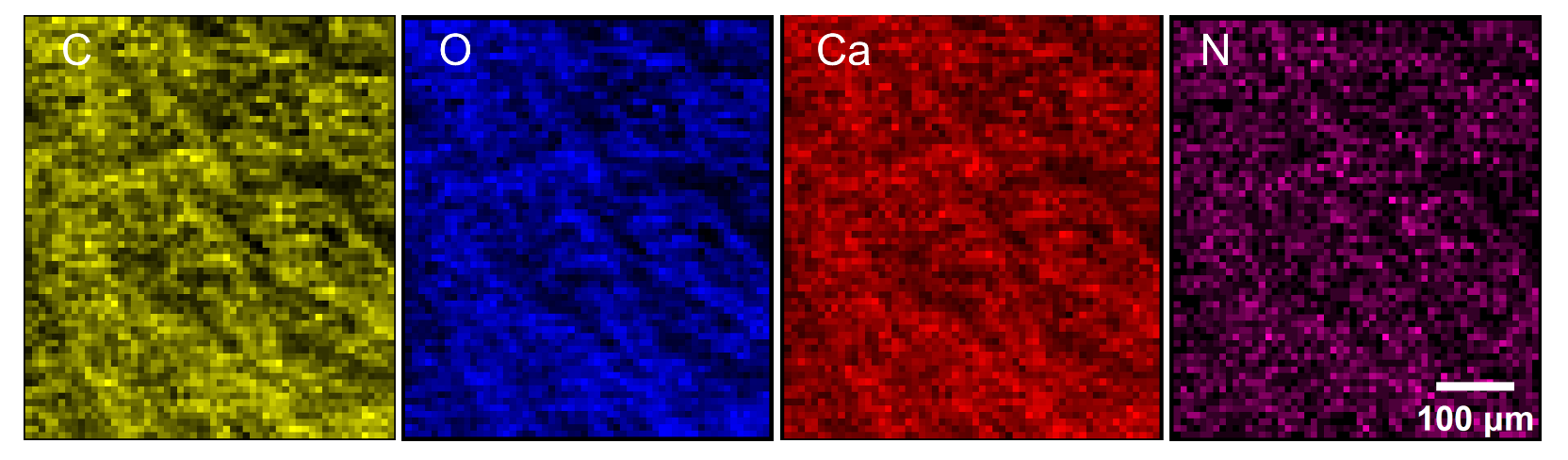


Figure S2. Element mapping of SMCC hydrogel.


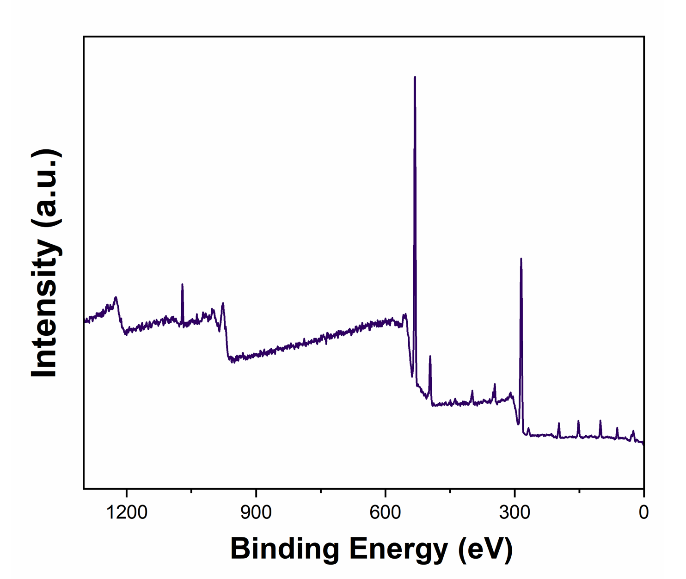


Figure S3. XPS full spectra of SMCC hydrogel.


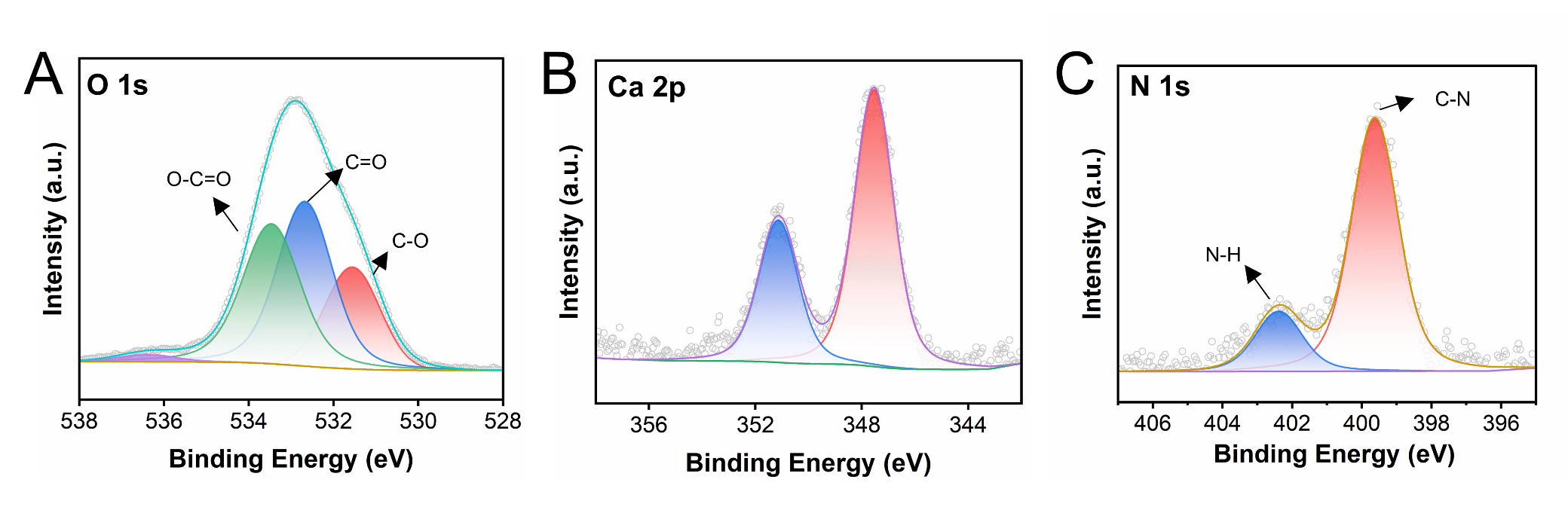


Figure S4. High-resolution A) O 1s, B) Ca 2p, and C) N 1s spectra of the SMCC hydrogel.


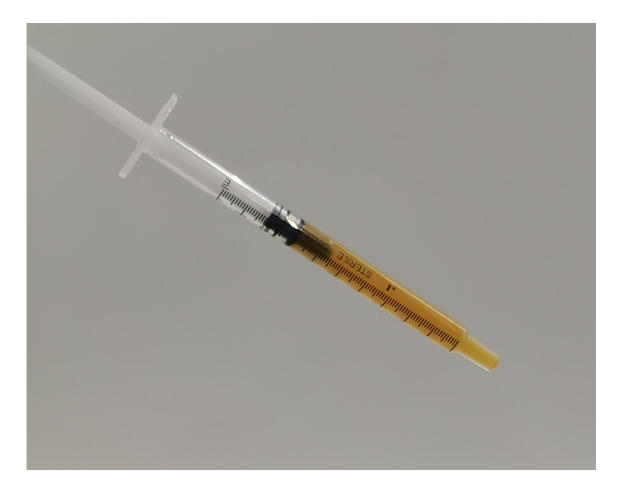


Figure S5. The injectability of SMCC hydrogel.


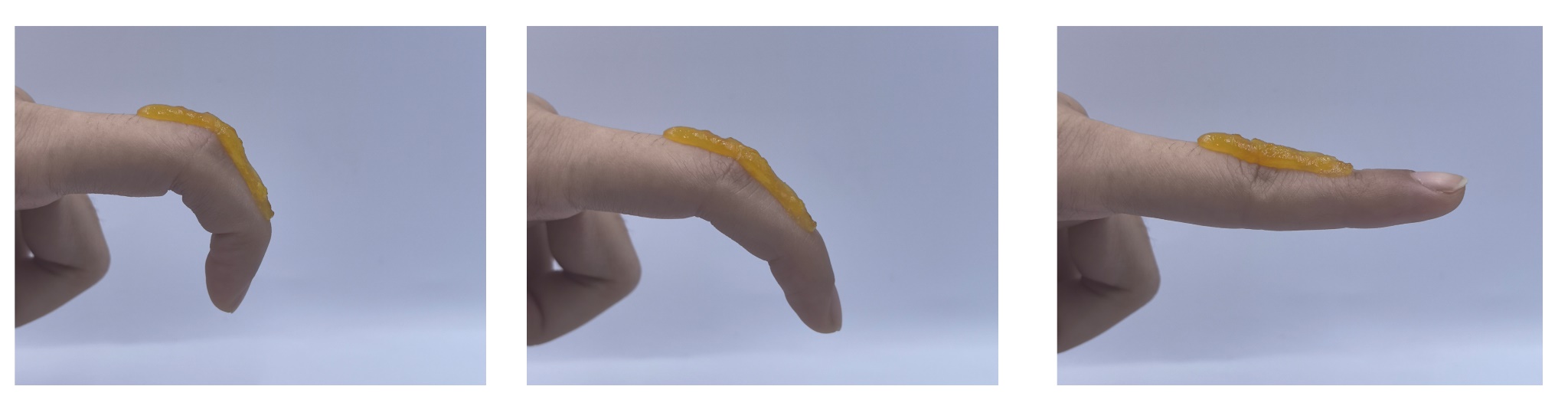


Figure S6. Dynamic adhesive behavior of SMCC hydrogel to the finger.


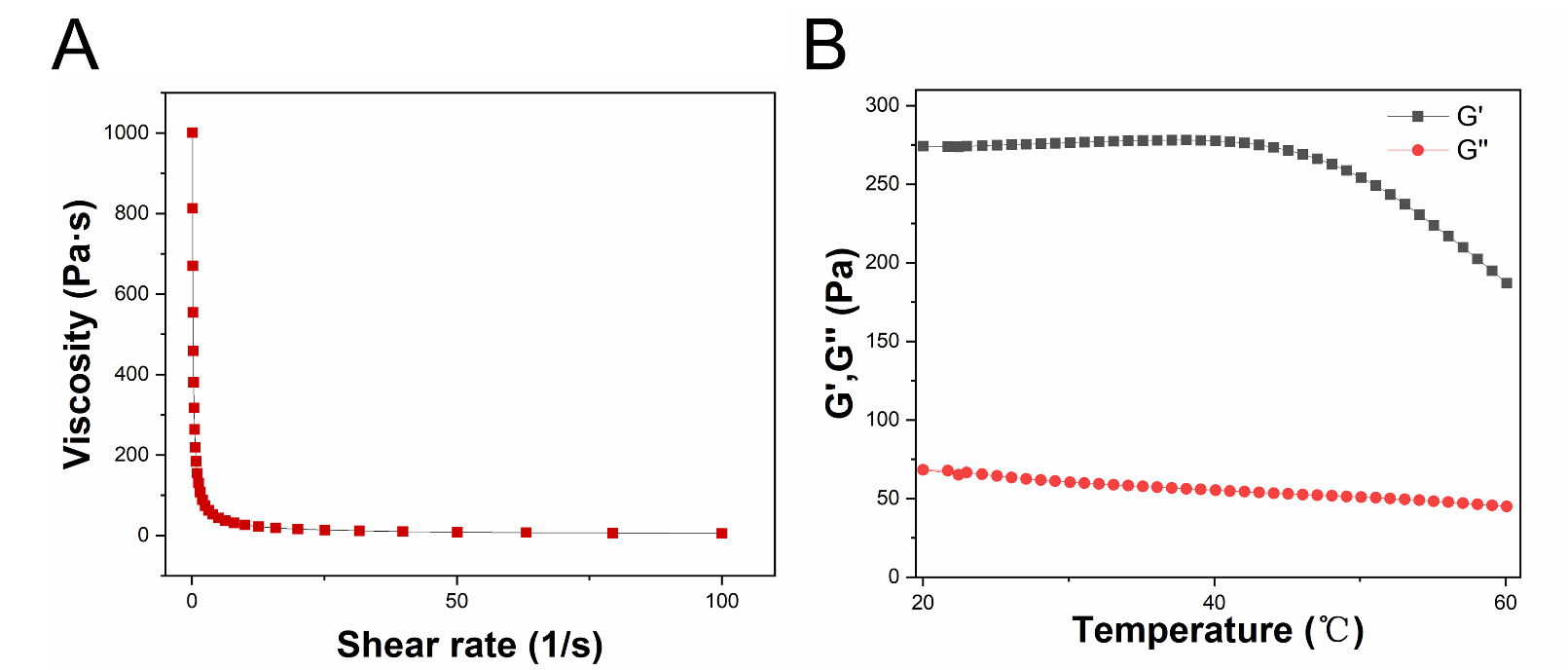


Figure S7. Rheological studies of SMCC hydrogel. A) Shear-thinning properties of SMCC hydrogel. B) Temperature sweep of SMCC hydrogel from 20 to 60℃.


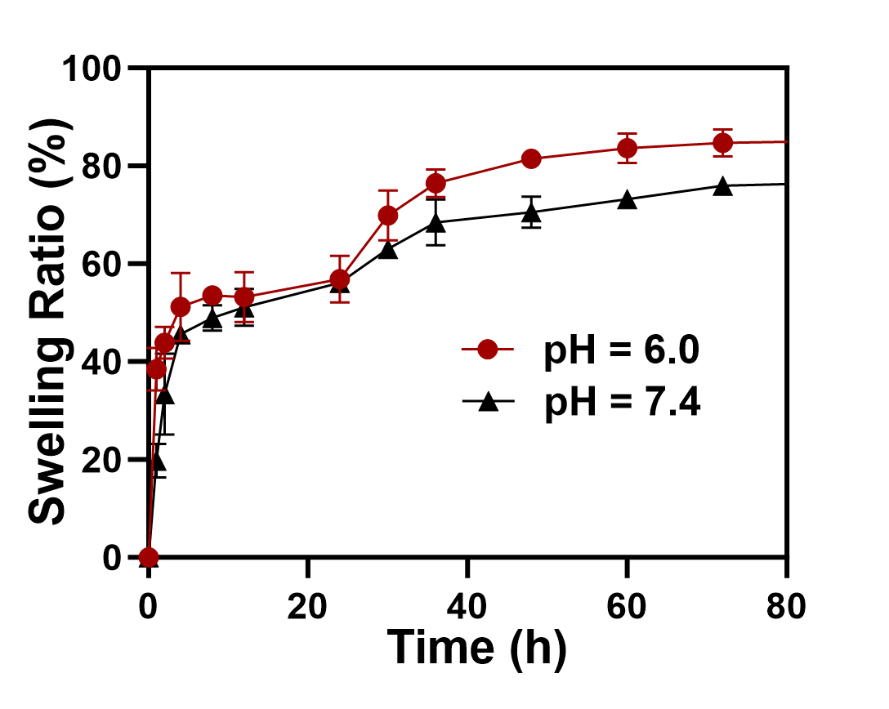


Figure S8. Swelling behavior of the SMCC hydrogel at different pH levels.


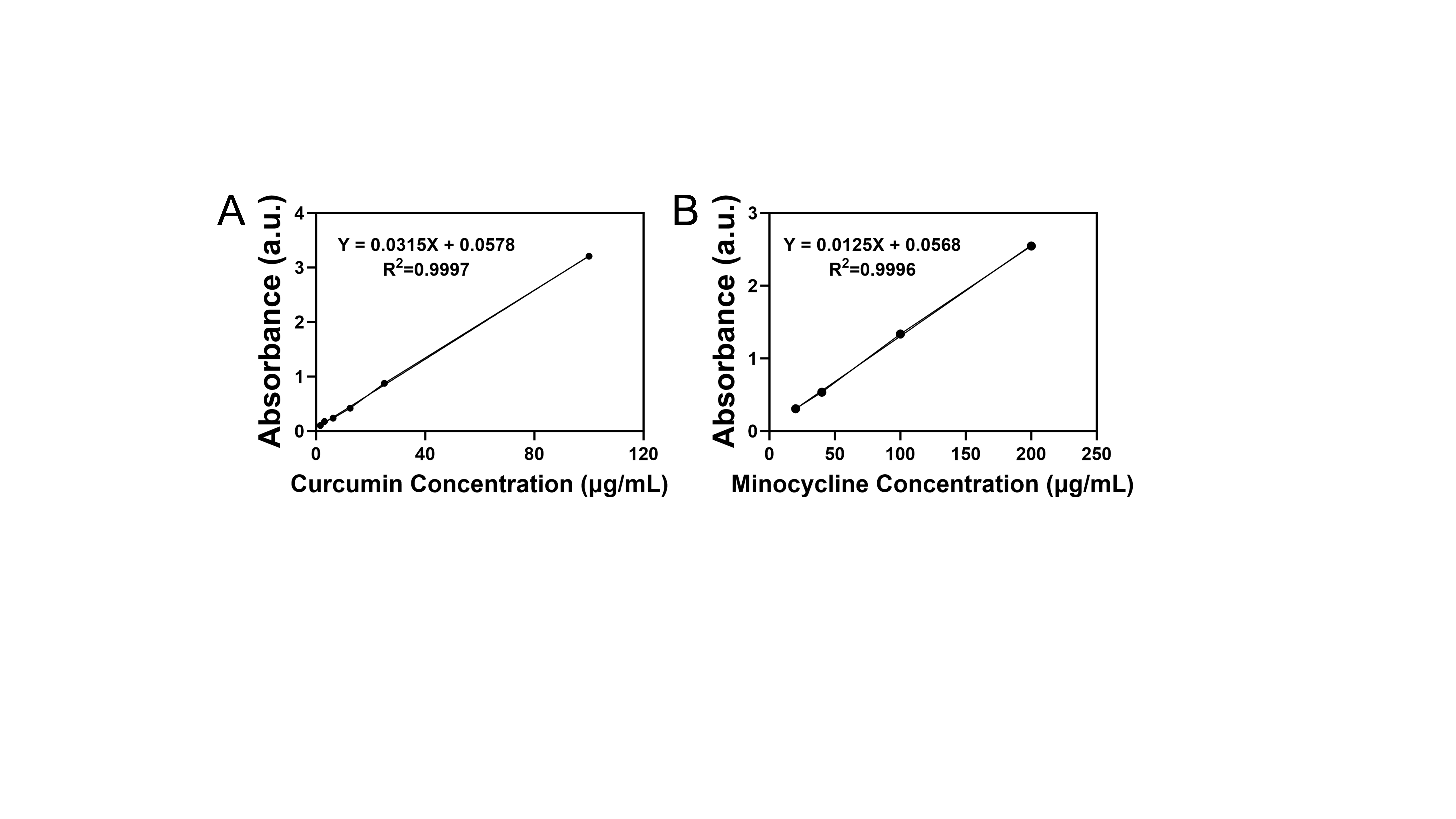


Figure S9. The standard calibration curve of Cur and Minocycline.


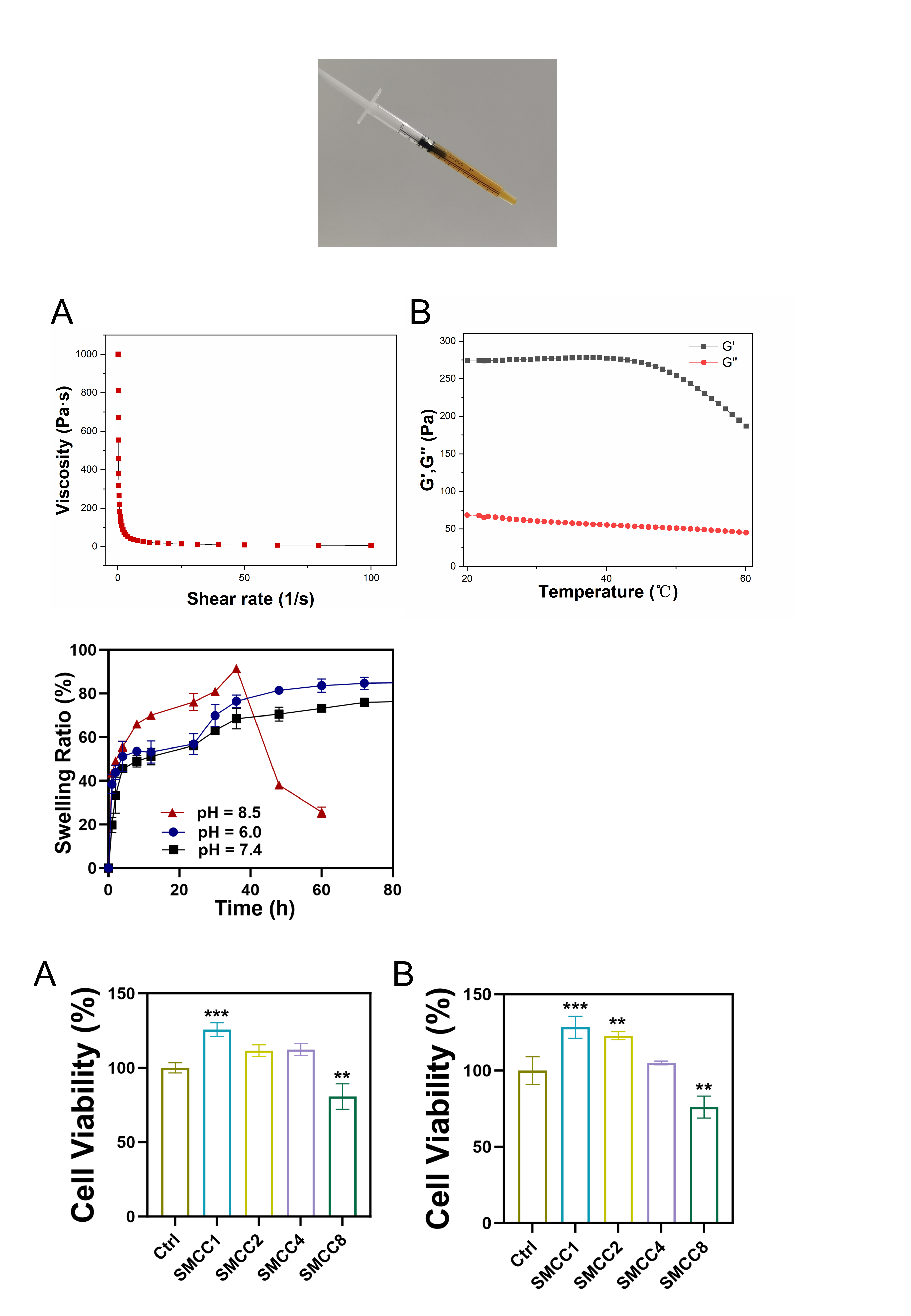


Figure S10. Cell viability experiment of RAW264.7 after 3 days of treatments (n=3).


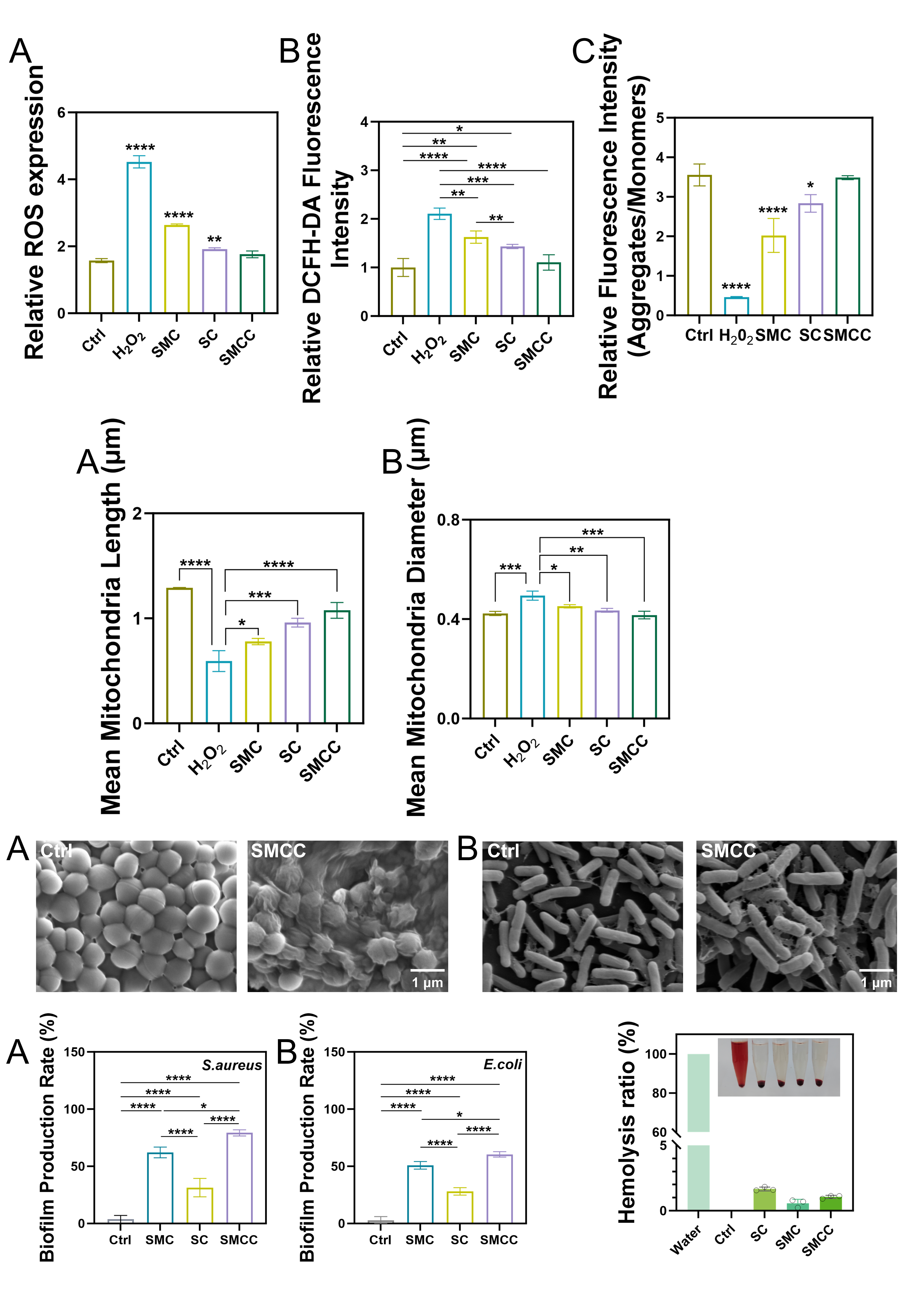


Figure S11. SEM images of A) *S. aureus* and B) *E. coli* treated by SMCC hydrogel.


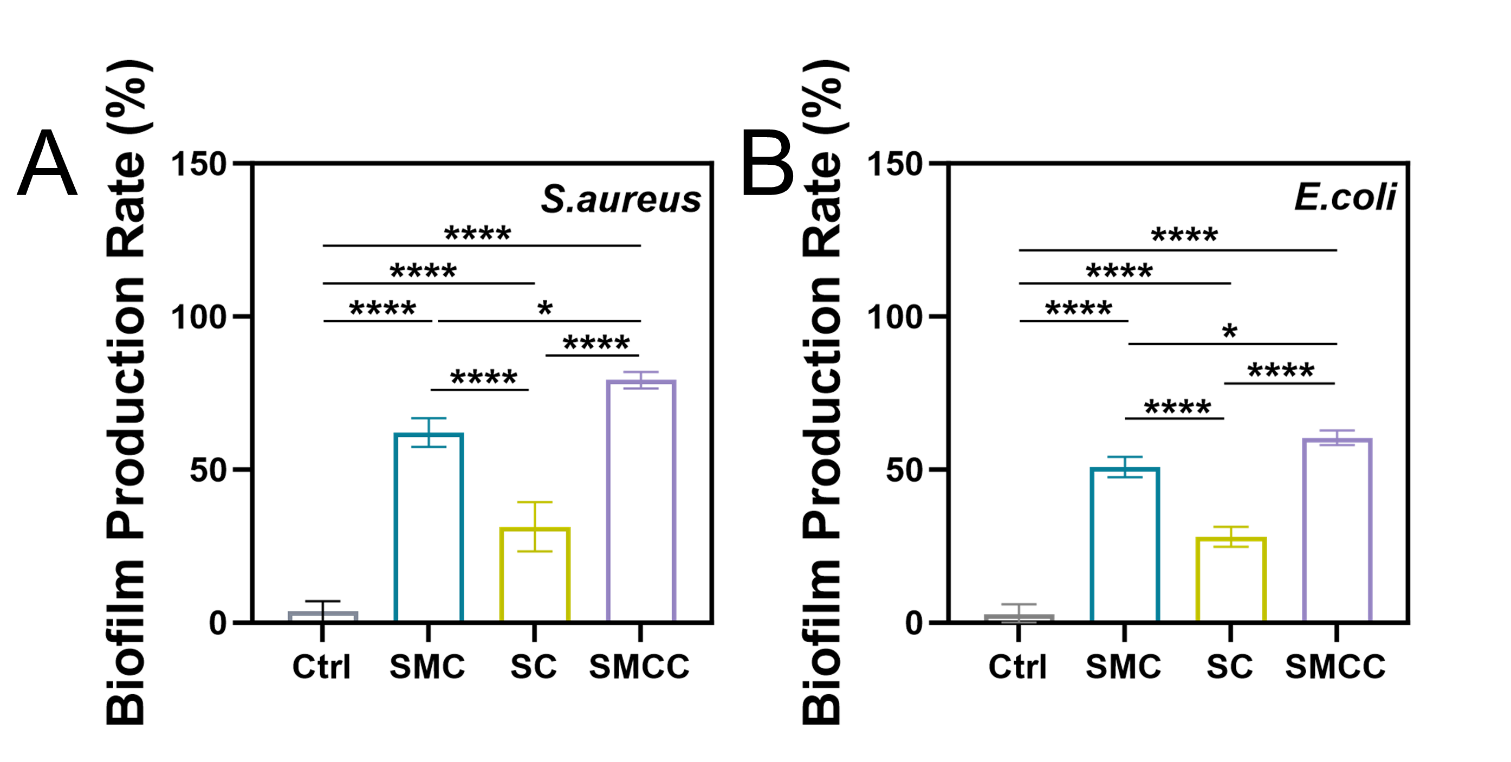


Figure S12. Quantified analysis of A) *S. aureus* and B) *E. coli* biofilms stained by crystal violet after treatments (n=3).


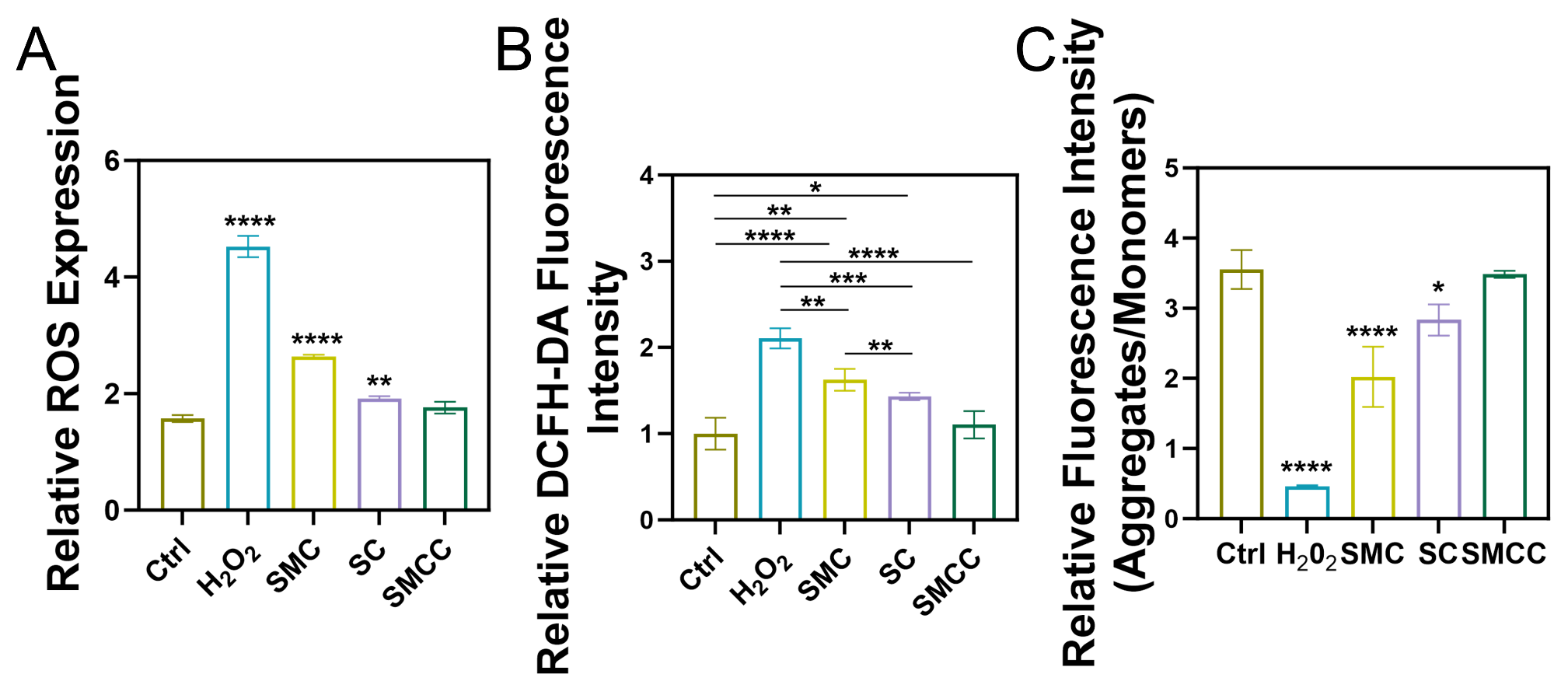


Figure S13. A) Quantified analysis of ROS flow cytometric. B) Fluorescence intensity of ROS in HUVECs (n=3). C) Quantitative analysis of JC-1 staining after treatments (n=3).


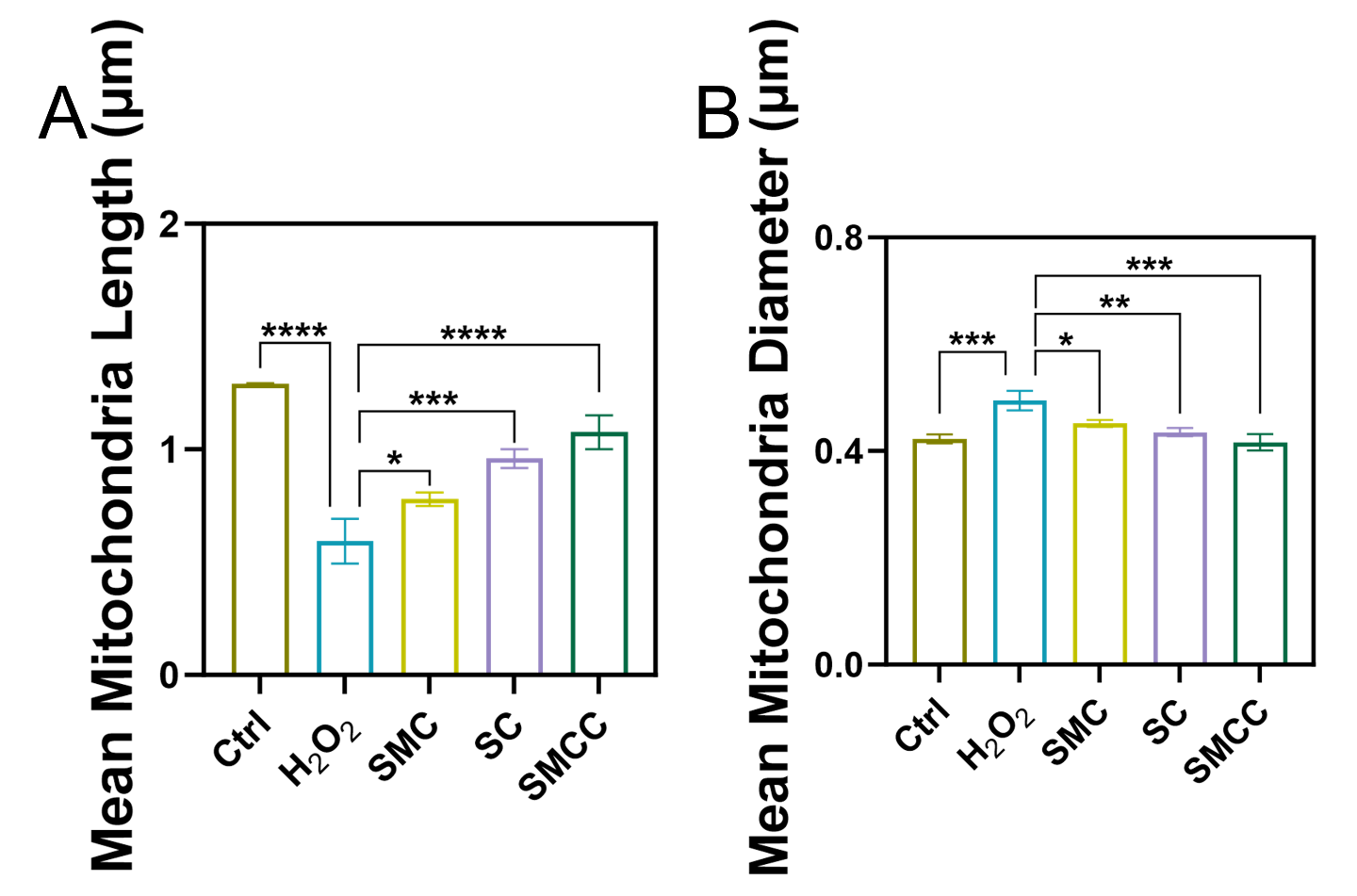


Figure S14. Mitochondrial morphological analysis of A) length and B) diameter after treatments (n = 3).


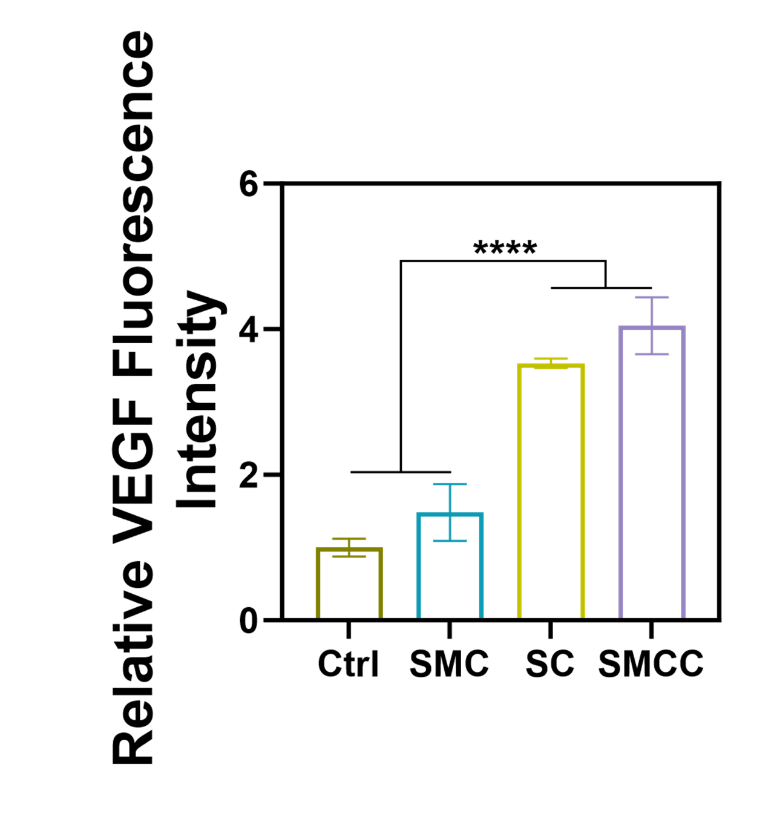


Figure S15. Quantitative analysis of VEGF staining after treatments (n=3).


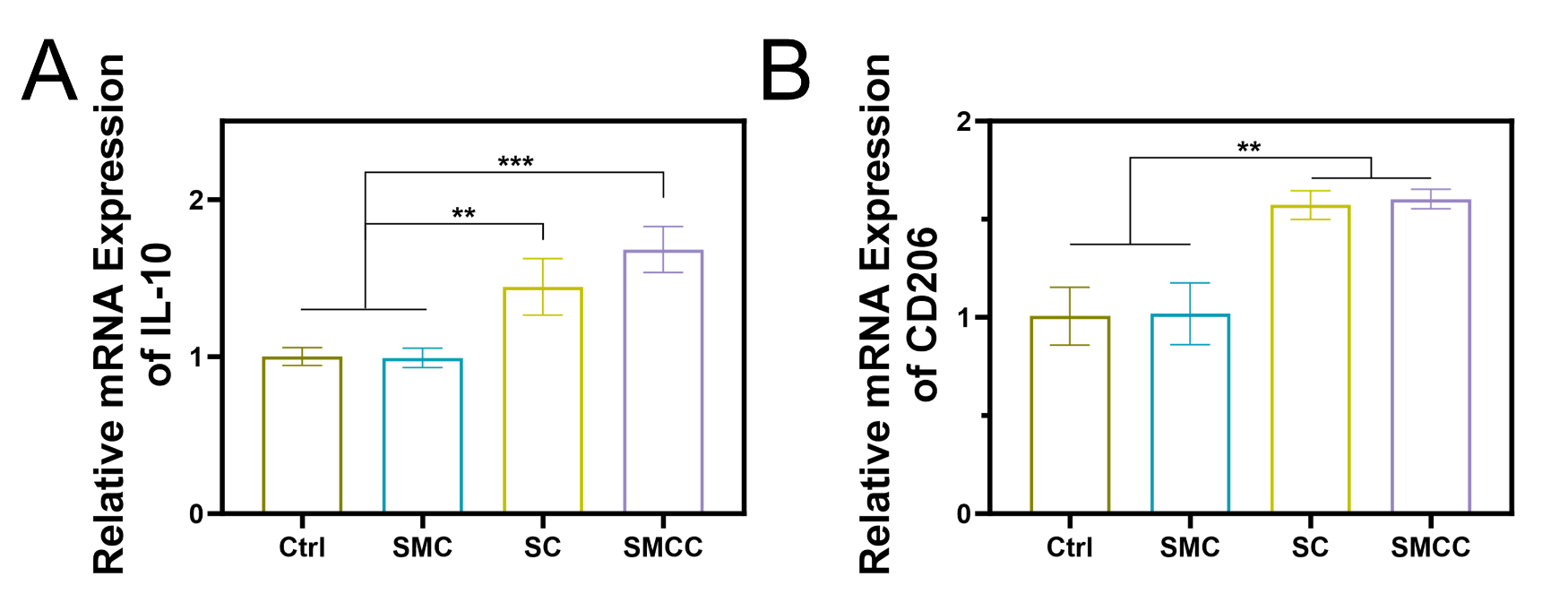


Figure S16. Expression of A) IL-10 and B) CD206 in macrophages assessed by RT-qPCR (n=3).


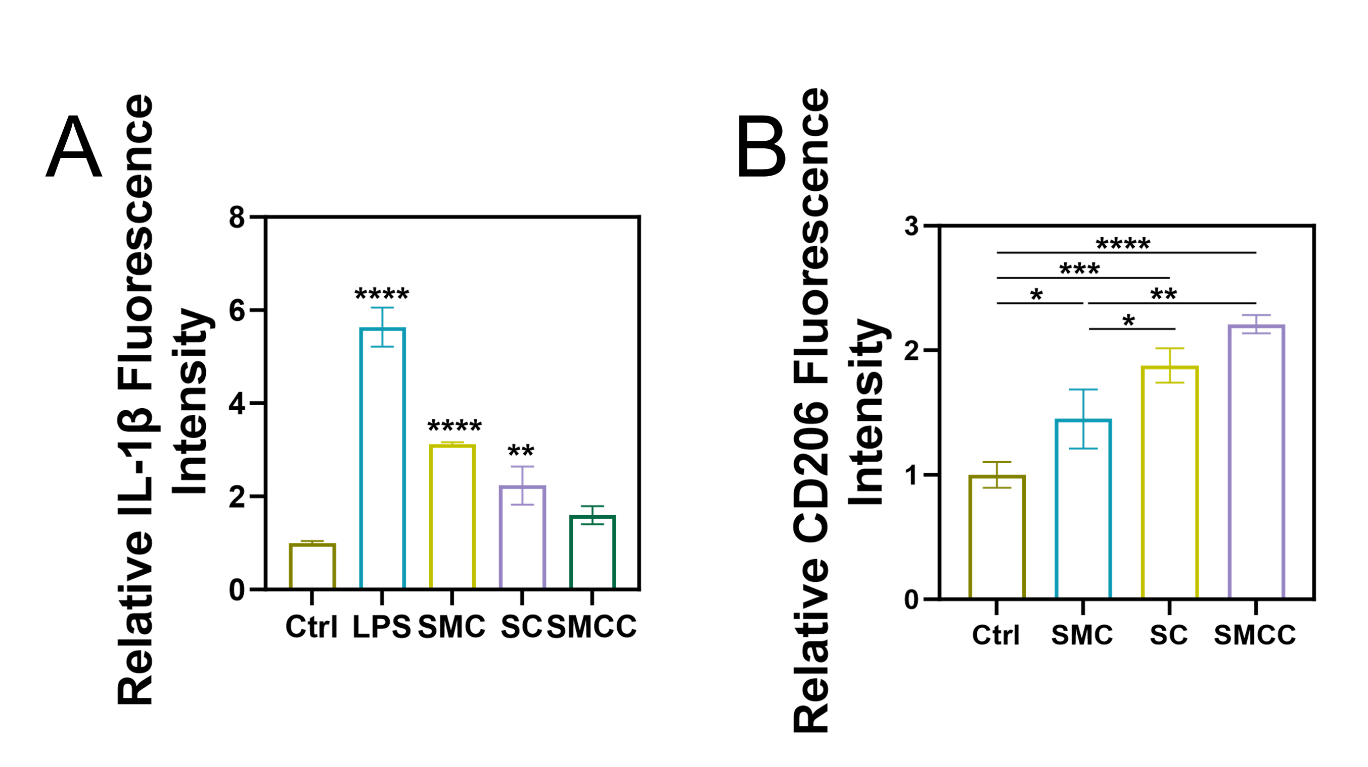


Figure S17. Quantitative analysis of A) IL-1β and B) CD206 staining after treatments (n=3).


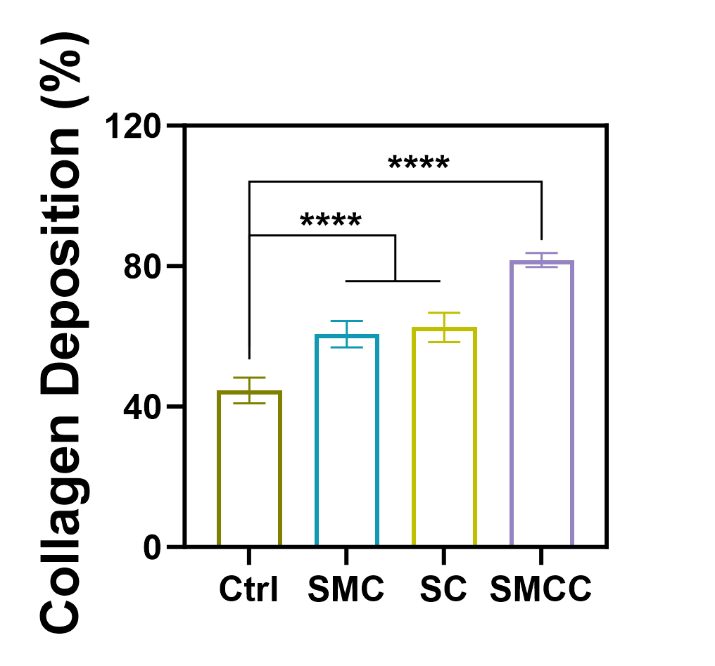


Figure S18.Quantification of collagen deposition on day 14 after different treatments (n = 5).


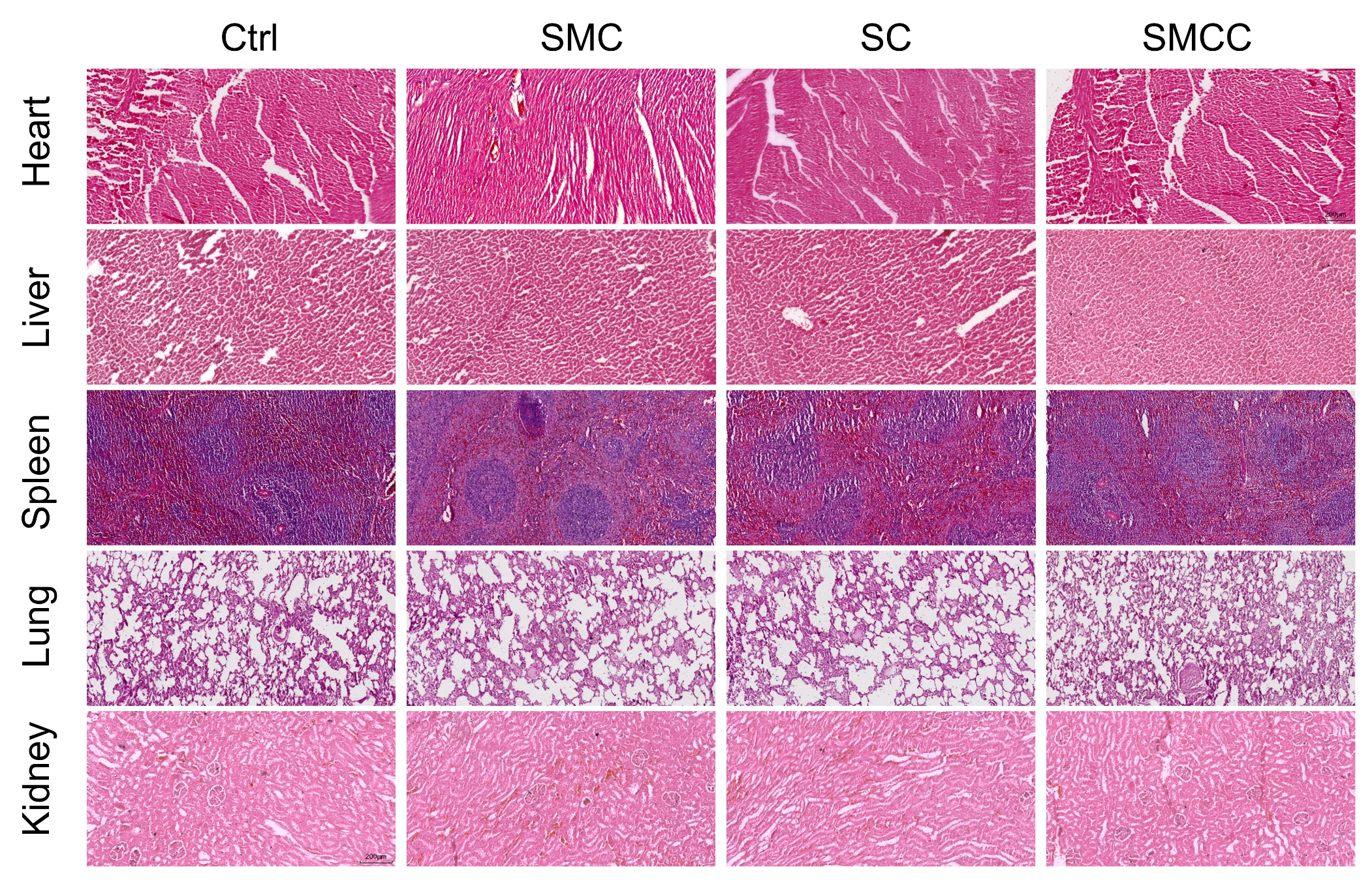


Figure S19. *In vivo* biocompatibility evaluation. H&E staining of the heart, liver, spleen, lung, and kidney.


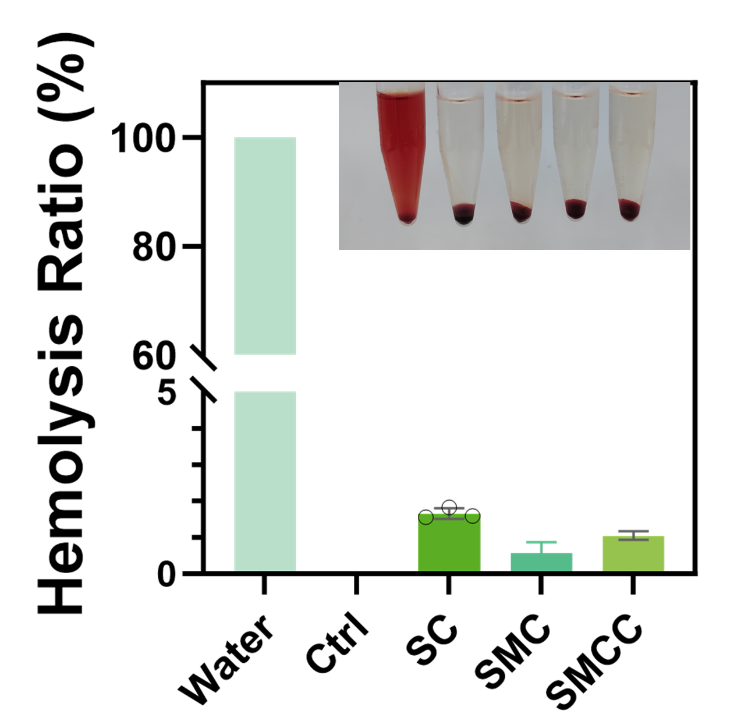


Figure S20. Diagram and hemolysis ratio of hydrogels.


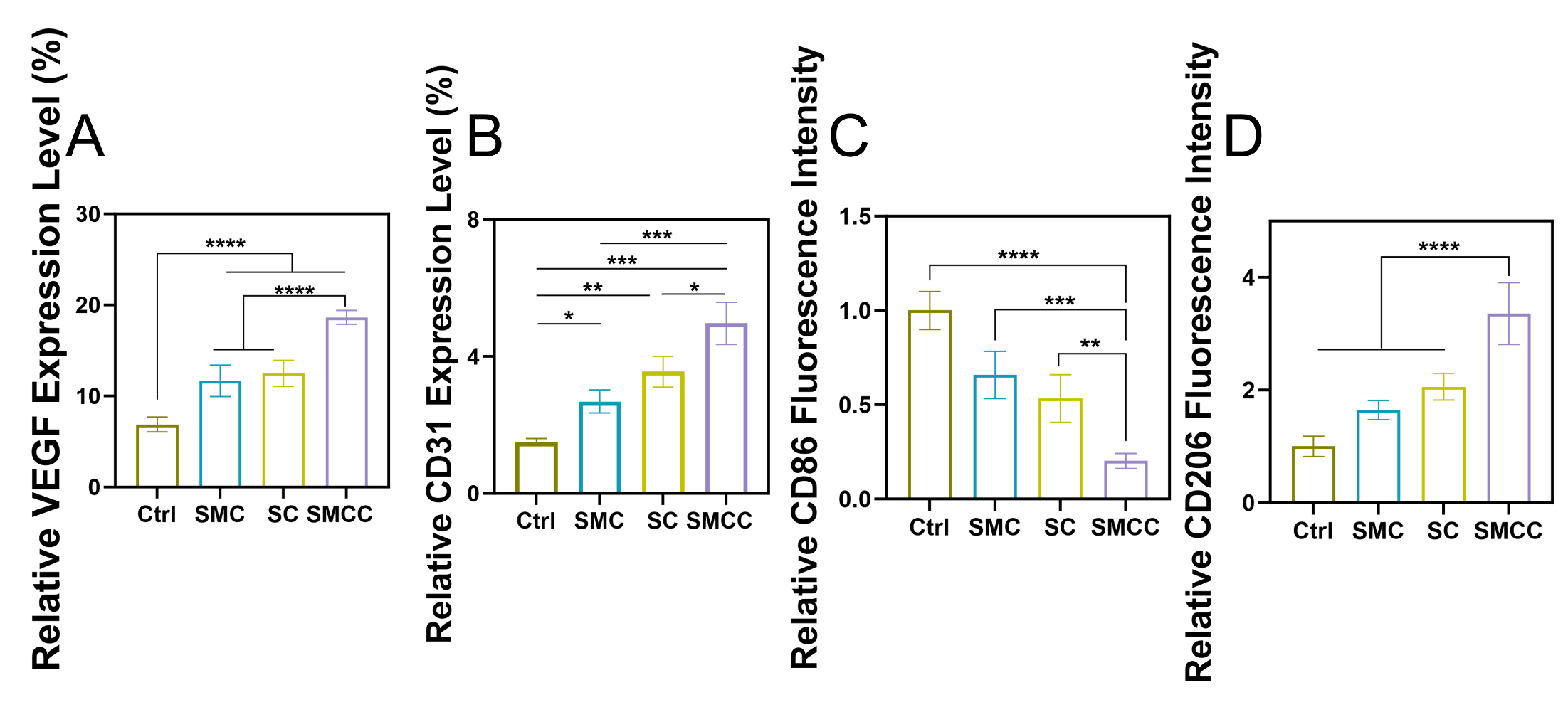


Figure S21. Statistical analysis of expression A) VEGF, B) CD31, C) CD86, and D) CD206 in wound sites after treatment (n=5).

**Table S1.** The abbreviation list.

| Abbreviation | Full name |
| --- | --- |
| SA | Sodium Alginate |
| MC | Minocycline hydrochloride |
| Cur | Curcumin |
| SMC | Sodium Alginate/Minocycline hydrochloride/Ca^2+^ Hydrogel |
| SC | Sodium Alginate/Curcumin/Ca^2+^ hydrogel |
| SMCC | Ca^2+^/Curcumin/Sodium Alginate/ Minocycline hydrochloride Hydrogel  (The concentration of Cur required for hydrogel preparation is 4 mg/mL) |

**Table S2.** The initial feeding composition of hydrogels.

|  | 5 wt% SA  water solution (mL) | MC powder (mg) | 1 wt% CaCl_2_  water solution (mL) | Cur solution |
| --- | --- | --- | --- | --- |
| SA | 4 | - | 1 | - |
| SMC | 4 | 2.5 | 1 | - |
| SC | 4 | - | 1 | 4.0 mg/mL, 1mL |
| SMCC1 | 4 | 2.5 | 1 | 1.0 mg/mL, 1mL |
| SMCC2 | 4 | 1 | 1 | 2.0 mg/mL, 1mL |
| SMCC4 | 4 | 2.5 | 1 | 4.0 mg/mL, 1mL |
| SMCC8 | 4 | 2.5 | 1 | 8.0 mg/mL, 1mL |

**Table S3.** Gene primer sequences used in the RT-qPCR experiments.

| Gene Name | Forward Sequences | Reverse Sequences |
| --- | --- | --- |
| m-IL-6 | TTCTTGGGACTGATGCTGGTGAC | GTGGTATCCTCTGTGAAGTCTCCTC |
| m-TNF-α | ACGCTCTTCTGTCTACTGAACTTCG | TGGTTTGTGAGTGTGAGGGTCTG |
| m-IL-1β | CTCGCAGCAGCACATCAACAAG | CCACGGGAAAGACACAGGTAGC |
| m-IL-10 | GGTTGCCAAGCCTTATCGGAAATG | GCCGCATCCTGAGGGTCTTC |
| m-CD206 | TGTACGCAGTGGTTGGCAGTG | GCTCTGATGATGGACTTCCTGGTAG |
| m-GAPDH | CCTTCCGTGTTCCTACCCC | GCCCAAGATGCCCTTCAGT |
| h-CD31 | TGACCCTTCTGCTCTGTTCAA | CTGAGGCTTGACGTGAGAGG |
| h-VEGF | GCAGAATCATCACGAAGTGGT | CCAGGGTCTCGATTGGATGG |
| h-α-SMA | ATGCCTCTGGACGCACAACT | CCCGGACAATCTCACGCTCA |
| h-GAPDH | GCACCGTCAAGGCTGAGAAC | TGGTGAAGACGCCAGTGGA |
| r-VEGF | TGAGACCCTGGTGGACATCT | GCTGGCTTTGGTGAGGTTTG |
| r-CD31 | AGTAGCATCCTGGTCAACATAACA | ACAACACCGTCTCTTCCTTCTG |
| r-α-SMA | GGGAGTGATGGTTGGAATGGG | CCGTTAGCAAGGTCGGATGC |
| r-CD206 | ACACACTGGAACGCTGACAT | TCCCATAAACCACCTGCCAC |
| r-IL-6 | ATTGTATGAACAGCGATGATGCAC | CCAGGTAGAAACGGAACTCCAGA |
| r-TNF-α | CACCATGAGCACGGAAAGCA | GCAATGACTCCAAAGTAGACC |
| r-GAPDH | ATGGTGAAGGTCGGTGTGAAC | GAGGTCAATGAAGGGGTCGTT |

[1] C. Xie, Q. Zhang, Z. Li, S. Ge, and B. Ma, "Sustained and Microenvironment-Accelerated Release of Minocycline from Alginate Injectable Hydrogel for Bacteria-Infected Wound Healing." Polymers 14(9) (2022): 1816. https://doi.org/10.3390/polym14091816.
